# A simple and efficient method to quantify the cell parameters of the seed coat, embryo and silique wall in rapeseed

**DOI:** 10.1186/s13007-022-00948-1

**Published:** 2022-11-03

**Authors:** Yushun Jiao, Baoling Liang, Guangsheng Yang, Qiang Xin, Dengfeng Hong

**Affiliations:** 1 National Key Laboratory of Crop Genetic Improvement, Huazhong Agricultural University, Wuhan, China; 2 Shanghai Key Laboratory of Plant Molecular Sciences, College of Life Sciences, Shanghai Normal University, Shanghai, China; 3 Hubei Hongshan Laboratory, Wuhan, China

**Keywords:** Rapeseed, Cell quantification, Cell size, High-throughput image processing

## Abstract

**Background:**

Researchers interested in the seed size of rapeseed need to quantify the cell size and number of cells in the seed coat, embryo and silique wall. Scanning electron microscope-based methods have been demonstrated to be feasible but laborious and costly. After image preparation, the cell parameters are generally evaluated manually, which is time consuming and a major bottleneck for large-scale analysis. Recently, two machine learning-based algorithms, Trainable Weka Segmentation (TWS) and Cellpose, were released to overcome this long-standing problem. Moreover, the MorphoLibJ and LabelsToROIs plugins in Fiji provide user-friendly tools to deal with cell segmentation files. We attempted to verify the practicability and efficiency of these advanced tools for various types of cells in rapeseed.

**Results:**

We simplified the current image preparation procedure by skipping the fixation step and demonstrated the feasibility of the simplified procedure. We developed three methods to automatically process multicellular images of various tissues in rapeseed. The TWS–Fiji (TF) method combines cell detection with TWS and cell measurement with Fiji, enabling the accurate quantification of seed coat cells. The Cellpose–Fiji (CF) method, based on cell segmentation with Cellpose and quantification with Fiji, achieves good performance but exhibits systematic error. By removing border labels with MorphoLibJ and detecting regions of interest (ROIs) with LabelsToROIs, the Cellpose–MorphoLibJ–LabelsToROIs (CML) method achieves human-level performance on bright-field images of seed coat cells. Intriguingly, the CML method needs very little manual calibration, a property that makes it suitable for massive-scale image processing. Through a large-scale quantitative evaluation of seed coat cells, we demonstrated the robustness and high efficiency of the CML method at both the single-cell level and the sample level. Furthermore, we extended the application of the CML method to developing seed coat, embryo and silique wall cells and acquired highly precise and reliable results, indicating the versatility of this method for use in multiple scenarios.

**Conclusions:**

The CML method is highly accurate and free of the need for manual correction. Hence, it can be applied for the low-cost, high-throughput quantification of diverse cell types in rapeseed with high efficiency. We envision that this method will facilitate the functional genomics and microphenomics studies of rapeseed and other crops.

**Supplementary Information:**

The online version contains supplementary material available at 10.1186/s13007-022-00948-1.

## Background

As the second most cultivated oilseed crop worldwide, rapeseed (*Brassica napus* L.) is one of the world’s most important sources of vegetable oil [1]. Rapeseed yield, a key concern of breeders and farmers, is characterized in terms of the number of siliques per plant, the number of seeds per silique and thousand seed weight. It has been reported that the seed weight shows an extremely significant correlation with the seed size in rapeseed [2]. The current understanding of the mechanisms controlling seed size reveals several signaling pathways that function through either maternal tissues (seed coat) or zygotic tissues (embryo, endosperm) [3]. As a protective tissue for the developing zygote, the seed coat orchestrates signal transduction between the endosperm/embryo and the external environment [4]. Additionally, the seed coat acts as a constraint and determines the seed size [5]. The embryo, as a zygotic tissue, can affect seed growth maternally by promoting cotyledon cell expansion or proliferation [6]. In addition to the seed parameters, the silique length also plays a major role in seed size regulation [7–10]. Silique wall cell elongation affects silique length and surface area and may lead to the accumulation of photosynthates and seed filling. In practice, researchers interested in seed size frequently need to observe the cell status and calculate the cell size and the number of cells in the seed coat, embryo, and silique wall.

The seed coat is derived from the outer and inner integuments of the ovule. In rapeseed, the mature seed coat consists of three layers: the epidermis/sub-epidermis, palisade and aleurone. As the most characteristic layer of the mature seed coat, the palisade is the main protective tissue [11]. The seed coat patterns determined by the palisade cells differ greatly among different brassica species [12]. Under scanning electron microscopy (SEM), the seed coat cell patterns can be recognized and used to measure cell size [13]. However, certain challenges hinder the application of this method, i.e., the seed margins of some materials are difficult to distinguish, and SEM assays are labor intensive and costly. As an alternative, bright-field images of seed coat cells can be acquired via a relatively simple procedure and applied for cell size measurement in rapeseed [2]. In both methods, however, the cell area is determined manually, which is time consuming and a major bottleneck for large-scale screening.

In fact, cell detection and quantitation are long-standing concerns of plant biologists [14–16]. Several automated and semiautomated measurement procedures have been developed to overcome this problem. For example, the cell-counter plugin in Fiji/ImageJ is widely used for counting cells of a certain area [16, 17]. Trainable Weka Segmentation (TWS), another plugin in Fiji, combines machine learning with manual annotation to train a classifier and process the remaining data automatically [18]. As a powerful tool for cell segmentation and object detection, TWS is widely applied in cell biology [19, 20]. Recently, a deep learning-based segmentation method called Cellpose was developed to deal with highly diverse images of cells without pretraining [21]. By combining the horizontal and vertical gradients predicted by a U-Net-shaped neural network, Cellpose generates vector fields from topological maps and assigns each pixel within a cell to a path converging at the center via gradient tracking [21]. Compared to other deep learning architectures, Cellpose achieves higher performance, especially on a previously unseen dataset [22]. Cellpose has been applied to segment myofibers within murine skeletal muscle with high accuracy and efficiency, even on complex images [23]. However, Cellpose generates labeled images that are difficult to process for users with no programming skills; therefore, some user-friendly plugins have been developed to solve this problem, including MorphoLibJ and LabelsToROIs [23, 24]. Although these advanced tools are powerful in cell segmentation, they have not been applied for cell quantification in rapeseed or other crops.

Here, we present a simple method to visualize the seed coat cells of mature and developing seeds, embryo cells, and silique wall cells. We also propose a cellular quantitation procedure based on Fiji and TWS/Cellpose for large-scale cell measurement. We have created several Fiji macros to harvest the cellular properties of the segmented cells automatically. Our method is a cost-efficient, labor-saving, and robust strategy that allows researchers to quantify the cell parameters of various types of cells in a high-throughput way.

## Results

### 1. Overview of the procedure for seed coat cell quantification

**Fig. 1:**
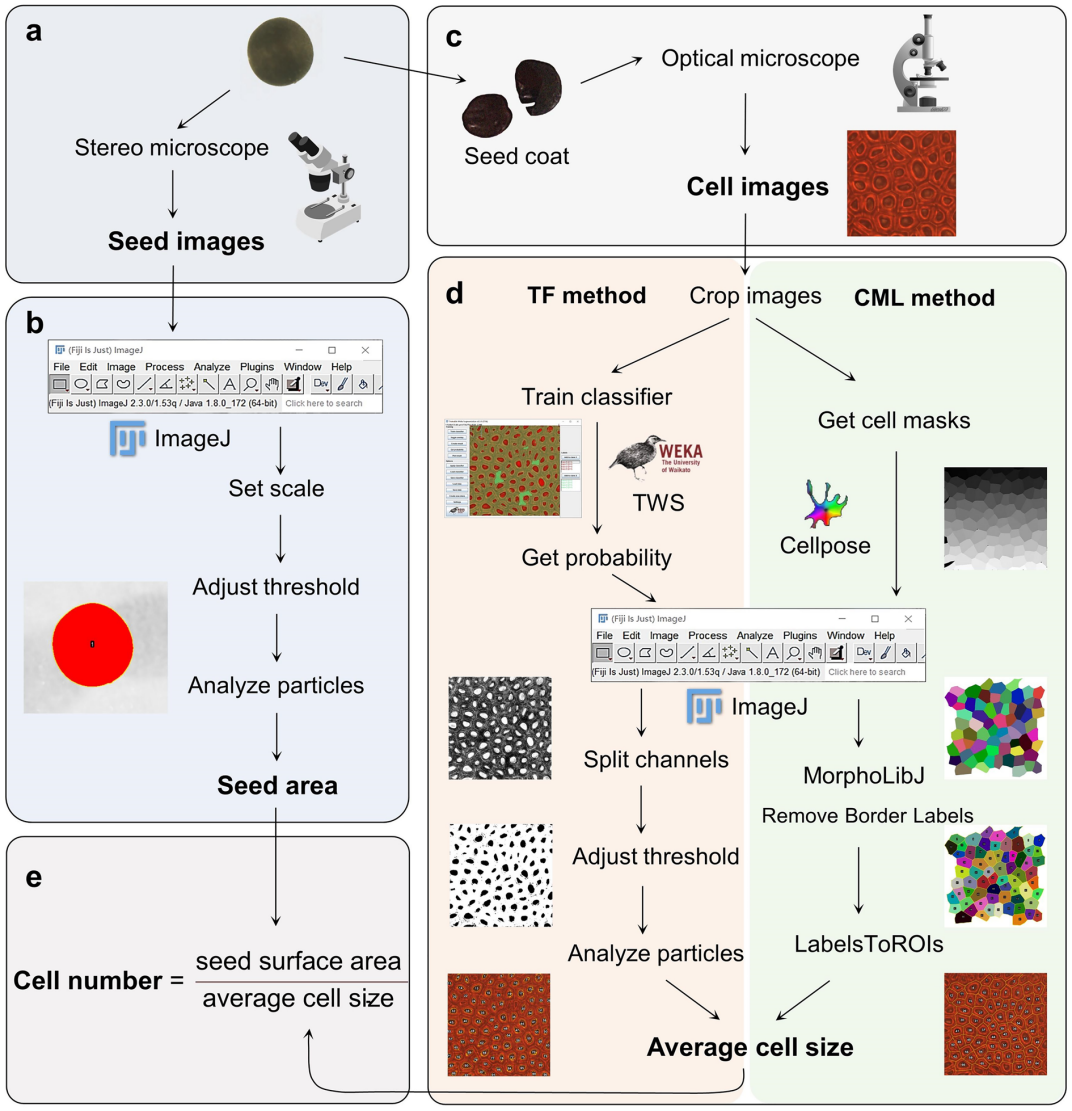
Flowchart of the procedure for quantifying seed coat cell parameters. **a** Seed images are acquired under a stereomicroscope. **b** The seed images are processed with Fiji software to calculate the seed area. **c** The seed coats are peeled off and photographed under an optical microscope. **d** The cell images are processed using the TWS–Fiji-based method (TF method) or the Cellpose–MorphoLibJ–LabelsToROIs-based method (CML method) to obtain the average cell size. Left, TF method; right, CML method; TWS, Trainable WEKA Segmentation. **e** The number of cells is calculated as the seed surface area divided by the average cell size

Here, we describe how to quantify the seed parameters of rapeseed, including the seed coat cell size and number of cells. The entire procedure consists of five modules. (i) The seeds are photographed individually under a stereomicroscope (Fig. 1a). (ii) The seed images are processed using Fiji software to obtain the seed area and the seed surface area (Fig. 1b). (iii) The seed coat is peeled off, treated to make it transparent, and photographed under an optical microscope to acquire seed coat cell images (Fig. 1c). (iv) The cell images are provided as input to the TWS–Fiji (TF) method or the Cellpose–MorphoLibJ–LabelsToROIs (CML) method to obtain the average cell size (Fig. 1d). (v) Finally, the number of seed coat cells is calculated as the seed surface area divided by the average cell size (Fig. 1e). Step (iii) is simplified by skipping the fixation step, and steps (ii) and (iv) can be automated; therefore, this procedure can be applied to high-throughput datasets and shows great potential for microphenomics studies.

### Acquisition of seed images and seed coat cell images

**Fig. 2:**
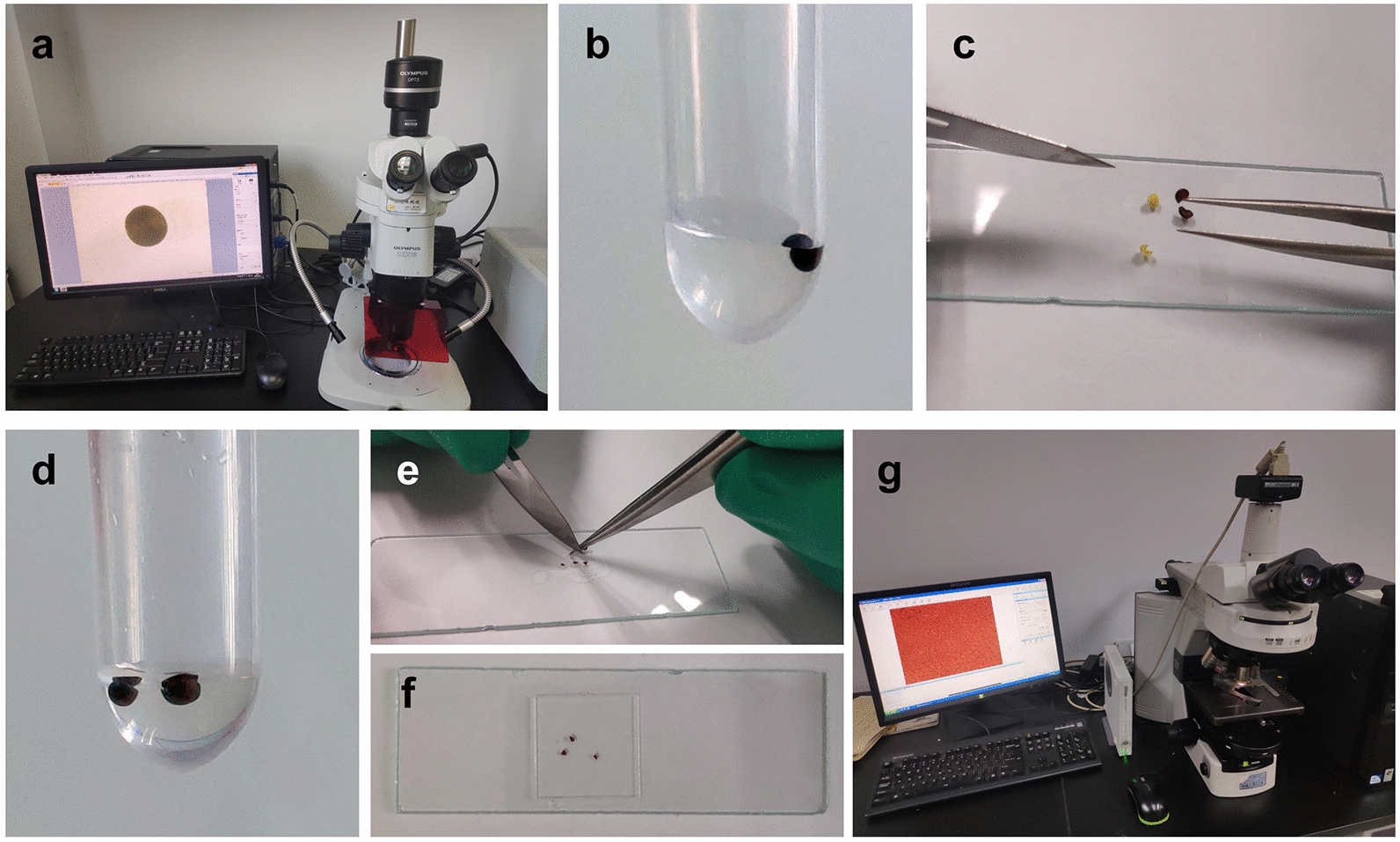
Flowchart of the acquisition of seed images and seed coat cell images. **a** Seeds were photographed under a stereomicroscope. **b** Each seed was submerged in distilled water. **c** Each seed was cut in half, and the seed coat was peeled off. **d** The seed coat was submerged in clearing solution. **e** The seed coat was cut into small pieces. **f** The seed coat pieces were prepared for photography. **g** Seed coat cells were photographed under an optical microscope

The commonly used method to measure seed coat cell size involves isolation of the seed coat, fixation with Formalin-Aceto-Alcohol (FAA) solution and a transparency procedure in chloral hydrate solution [2]. Considering that by the end of maturation, the cells of palisade layer of seed coat have all died but their cell walls remain [25], it is reasonable to postulate that the fixation of the cell structure can be skipped. First, we chose fully grown mature seeds of rapeseed and photographed each seed under a stereomicroscope to calculate the seed area/diameter and the seed surface area (Fig. 2a, Additional file 1). Second, we let the seed imbibe and peeled off the seed coat (Fig. 2b, c). Third, we cut the seed coat in half and submerged it in clearing solution (Hoyer’s solution) to make it transparent (Fig. 2d). Then, a small piece in the equatorial cross-sectional area of each half was cut and prepared for optical microscope observation (Fig. 2e–g). Although the fixation step was skipped, we obtained clear, high-quality cell images that were adequate for further analysis (Additional file 2).

### Seed size measurement of mature seeds with Fiji

**Fig. 3:**
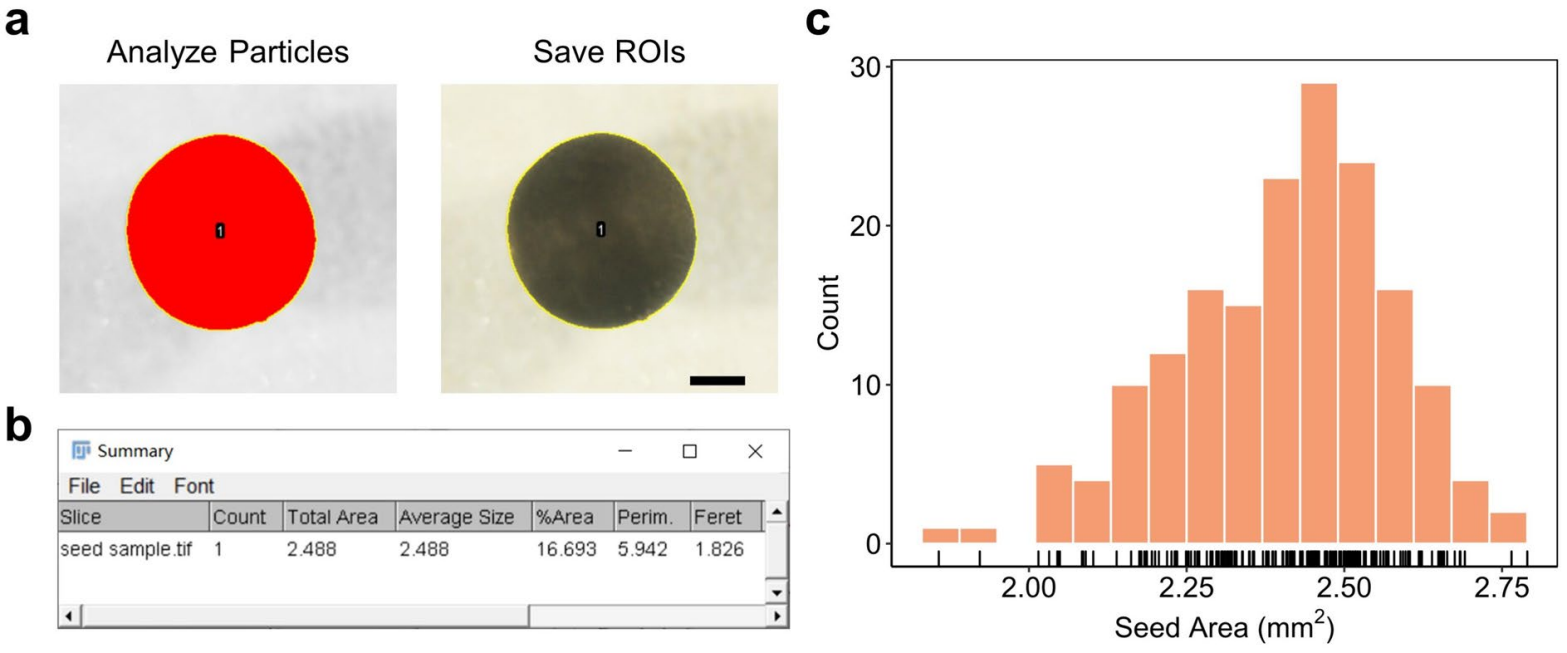
Seed area measurement with Fiji. **a** Seed images were processed with the “Analyze particles” function in Fiji. Bar = 0.5 mm. **b** Summary window of Fiji. The seed area was identified from the seed image in **a**. **c** Frequency distribution analysis of the seed area (n = 172)

The black seeds photographed under the stereomicroscope could be easily distinguished from the white background (Additional file 1). By adjusting the threshold in Fiji, we could precisely identify the seed outlines. The seed area was calculated by analyzing particles and visualized by adding the ROIs (region of interest) (Fig. 3a). The results were summarized in a new window (Fig. 3b). To test the robustness and large-scale detection ability of our method, we chose 172 mature dry seeds of rapeseed and measured their seed area. We created a macro to perform this task automatically and acquired results within minutes (Additional file 3). The average seed area was 2.40 mm^2^ with a range from 1.86 to 2.79 mm^2^ (Fig. 3c, Additional file 4: Dataset S1). The results demonstrate that the Fiji software is efficient and robust for measuring the seed size of rapeseed from raw images.

### Seed coat cell quantification of mature seeds based on Trainable Weka Segmentation (TWS)

We obtained high-quality images of the seed coat cells, which showed a red background due to the pigmentation of the palisade layer. The palisade cell walls and cell cavities were clearly observed under a 400 × microscope (Additional file 2). Overall, the cells showed polygonal shapes and large differences in size.

**Fig. 4:**
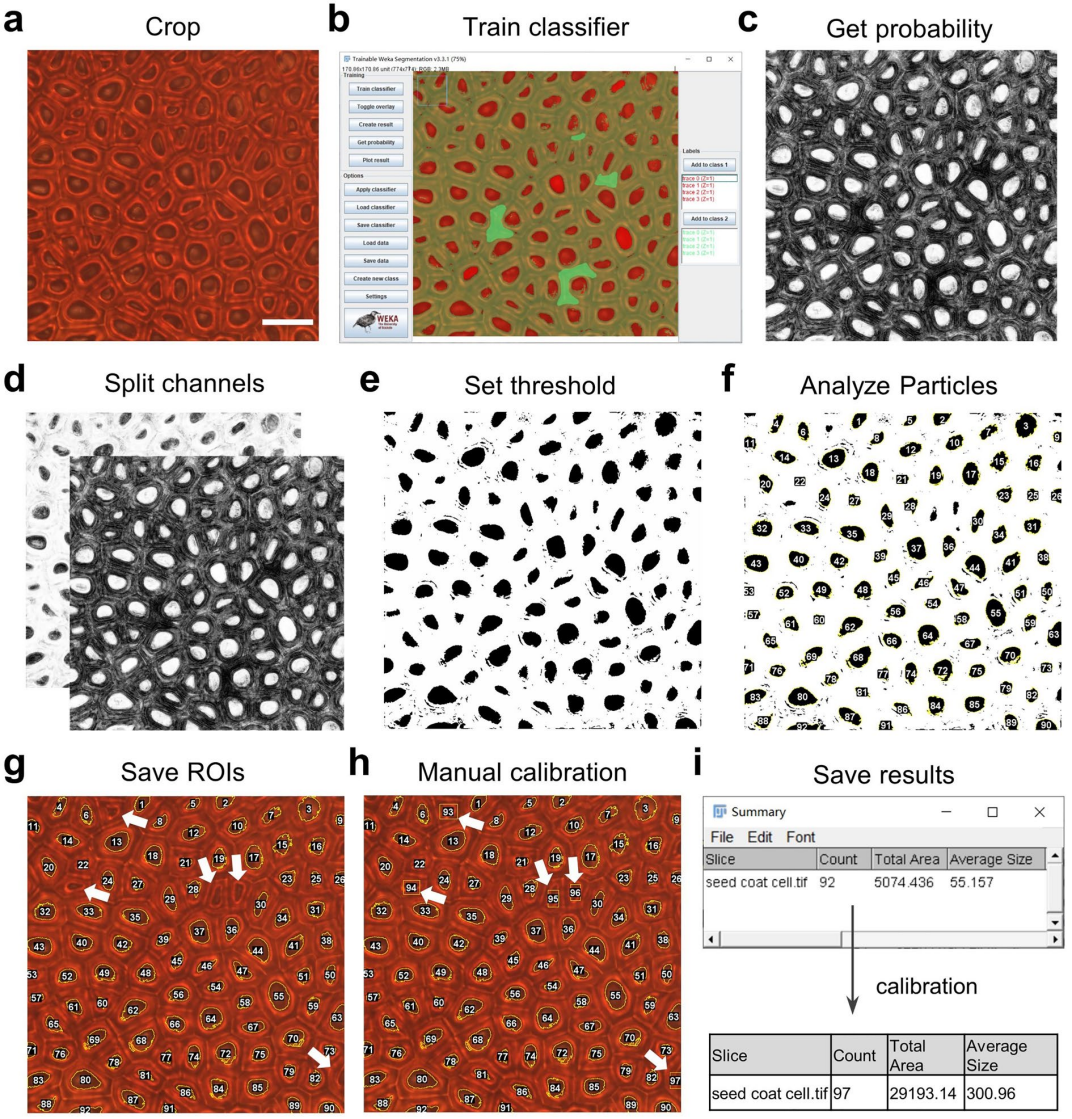
Seed coat cell size measurement of mature seeds via the TF method. **a** A raw cell image was randomly cropped. Bar = 30 μm. **b** The cropped cell image was input into Trainable Weka Segmentation, and a classifier was trained to segment the cell cavities and cell walls. **c** A probability map was acquired. **d** The two channels were split, and the cell cavity channel was further processed. **e** A threshold was set automatically. **f** The cells were counted by analyzing particles. **g** ROIs were added to the original image. The arrows indicate missing cells**. h** The ROIs were manually calibrated. The arrows indicate missing cells labeled manually. **i** The cell counts before and after calibration were saved, and the cell size was calculated. Image **a** is provided in Additional file 6

The first idea for measuring the cell size was to divide a certain area by the number of cells in this area. We tried to segment the cells with TWS and count the cells with Fiji. This method is designated the TF method for short. The seed coat cell images were randomly cropped to retain ~100 cells (Fig. 4a), which were considered sufficiently representative of the whole seed. We cropped an area of 29,193.14 μm^2^ from an image of seed coat cells and attempted to count the cells in this area. The cropped image was input into the TWS plugin in Fiji. We drew freehand selections around the seed cavity and wall regions and added them to classes 1 and 2, respectively, and then trained a classifier for cell cavity detection (Fig. 4b). Then we applied the classifier and obtained probability maps (Fig. 4c). The cell cavity channel was split from the two probability map channels and used for further analysis (Fig. 4d). By setting an appropriate threshold and analyzing particles, 92 cells were successfully identified (Fig. 4e, f). The ROIs were added to the original image for further inspection. Interestingly, although most of the cells could be correctly annotated, there were still 5 cells missing (Fig. 4g). We performed a manual calibration and eventually identified 97 cells. By dividing this area by the number of cells, we calculated the average cell area as 300.96 μm^2^ (Fig. 4h, i). We applied the TF method to another 6 images of seed coat cells and found that the average precision (AP) of cell detection reached 98.2% (Additional file 5: Fig. S1). These results reflect the feasibility and reliability of the TF method.

### Seed coat cell size measurement of mature seeds based on Cellpose

We also attempted to segment individual cells by using Cellpose and then measure the area of each cell. We first attempted to segment the cells with Cellpose and calculate the cell size with Fiji. This method, which is called the CF method, is summarized in Additional file 7: Fig. S2a. A cropped image of seed coat cells was input into the Cellpose algorithm for segmentation (Additional file 7: Fig. S2b). The cell mask file was then exported and processed further in Fiji (Additional file 7: Fig. S2c). By finding edges and setting the threshold to (0, 0), the cells were identified successfully (Additional file 7: Fig. S2d, e). Next, we performed cell area measurement using the “Analyze particles” plugin and found 70 cells, excluding the cells on the edges (Additional file 7: Fig. S2f). Furthermore, we added ROIs to the original image and obtained the cell parameters of each cell (Additional file 7: Fig. S2g, h, Additional file 4: Dataset S8). Despite the highly accurate cell segmentation, we consistently found small gaps between neighboring cells (Additional file 7: Fig. S2i), which occurred in the “Find edges” step and may cause bias in the cell areas.

**Fig. 5:**
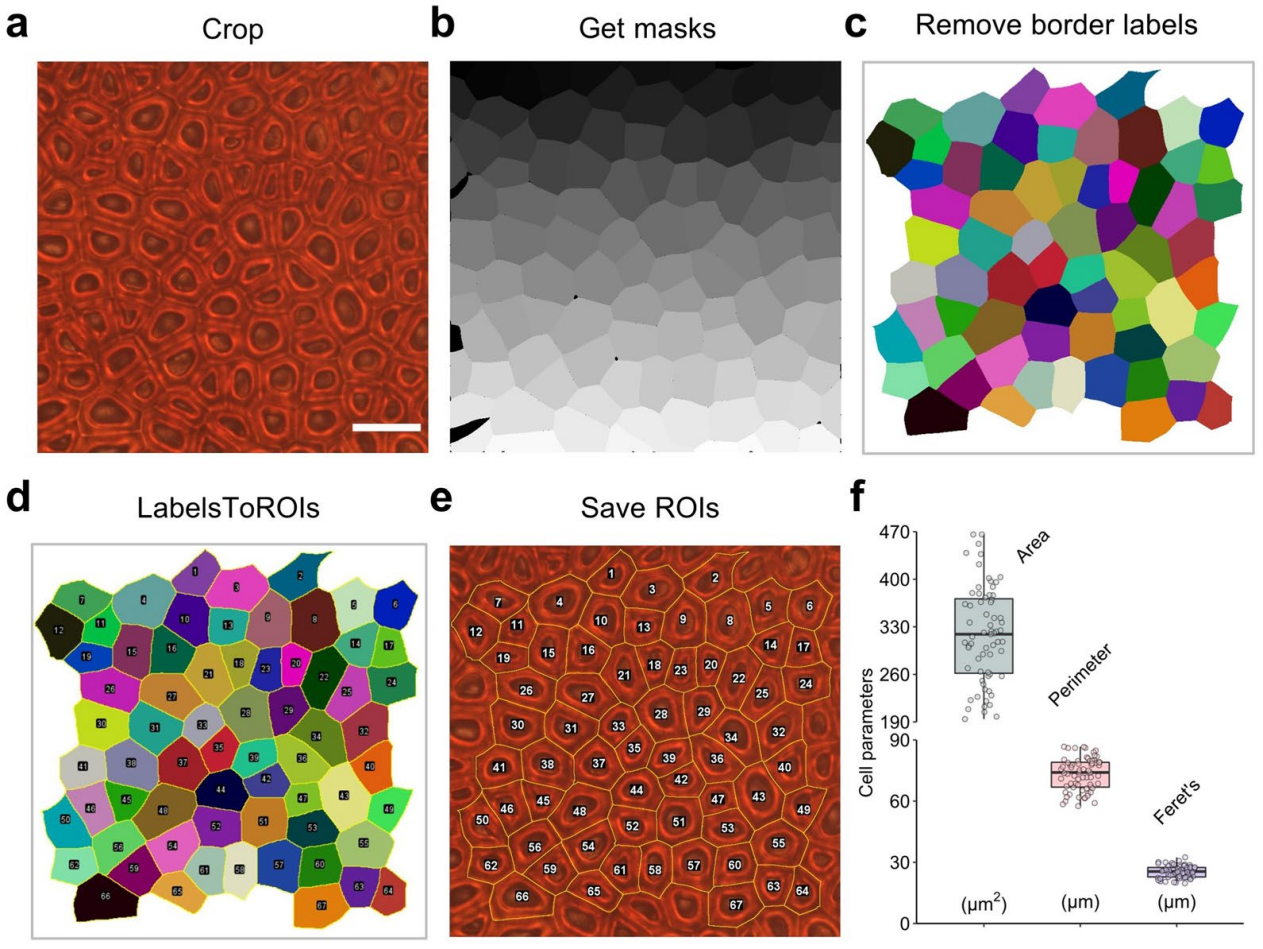
Seed coat cell size measurement of mature seeds via the CML method. **a** A raw cell image was randomly cropped. Bar = 30 μm. **b** The cell mask was acquired using Cellpose. **c** The border labels were removed by the MorphoLibJ plugin. **d** The label information was transformed into ROIs by the LabelsToROIs plugin. **e** The ROIs were saved for future inspection. **f** The cell parameters were acquired from image **a**. The boxplots represent the median and the 25th and 75th quartiles; the whiskers represent the minimum and maximum (n = 67). Images **a** and **b** are provided in Additional file 6 and 8

To circumvent this bias, we tested another two plugins in Fiji, MorphoLibJ and LabelsToROIs, which were developed to handle labeled images in a user-friendly manner. We call this method the CML method. First, a cropped image of seed coat cells was segmented by Cellpose to generate a cell mask file (Fig. 5a, b). Then, the mask file was fed to MorphoLibJ to remove border labels and extremely small labels (Fig. 5c), and was then passed to LabelsToROIs to transform the label information into ROIs (Fig. 5d). We saved the ROIs to the original file (Fig. 5e), and found nearly perfect cell segmentation results, with highly accurate detection of the cell margins. The parameters of the individual cells were saved to a csv file. The average cell size was 318.17 μm^2^, with a wide range from 194 to 466 μm^2^ (Fig. 5f), which partially explained the difficulty of quantifying the seed coat cell size. The average perimeter and Feret’s diameter were 73.16 μm and 25.34 μm, respectively (Additional file 4: Dataset S2). Defined as the longest distance between any two points along a boundary, Feret’s diameter is an important characteristic for describing polygons. Unlike in the CF method, there were no gaps between adjacent cells, indicating that the CML method is more accurate. These results reveal that the CML method is practical and precise for seed coat cell quantification.

### *Comparison of the cell parameters calculated *via* the TF, CF and CML methods*

**Fig. 6:**
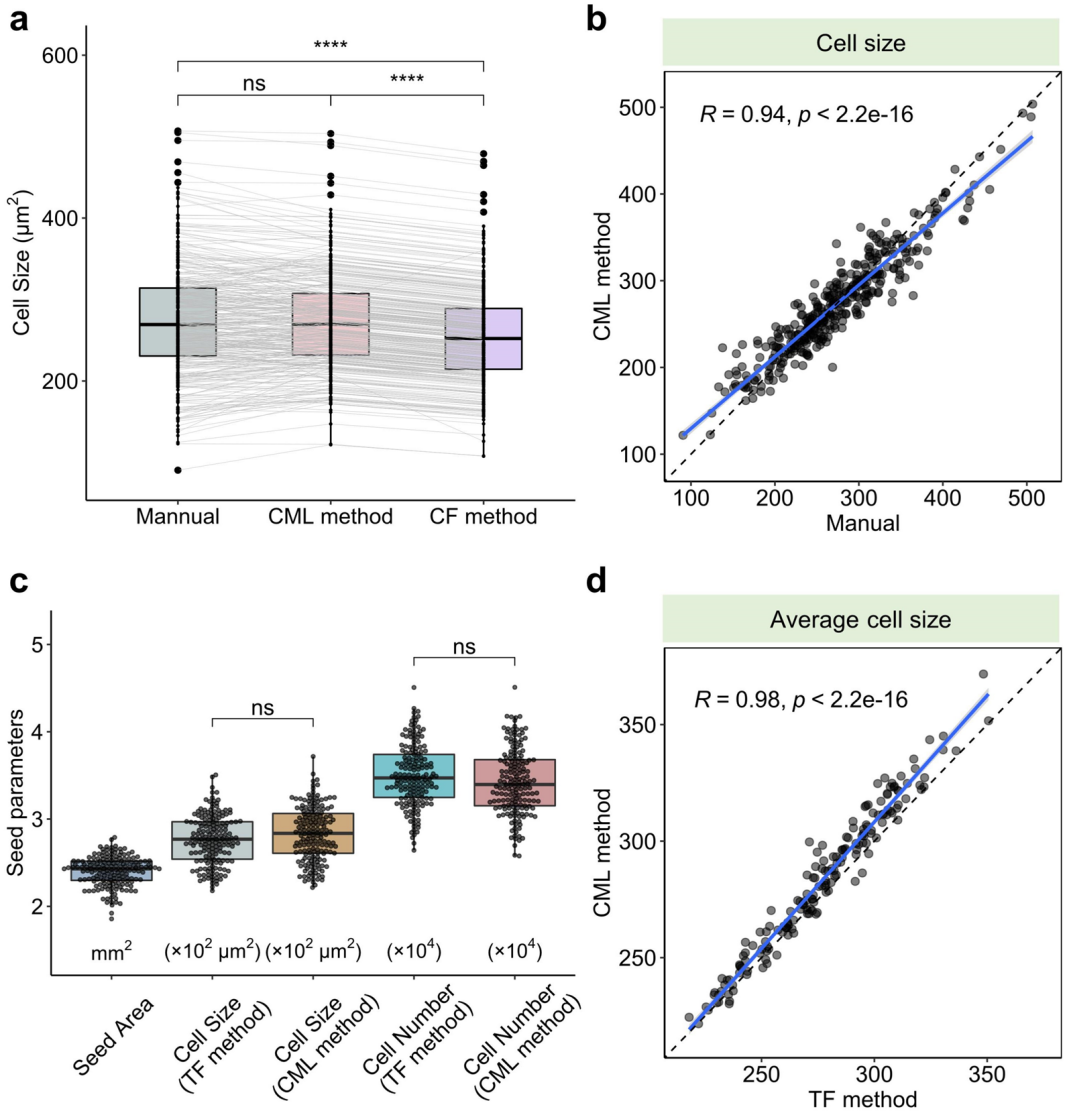
Comparison of seed parameters calculated via different methods. **a** Individual cell areas calculated via the manual, CF and CML methods (n = 362). **b** Correlation analysis (Pearson, confidence interval = 95%) between the individual cell sizes calculated via the manual and CML methods (n = 362). **c** Seed parameters calculated via the TF and CML methods (n = 172). **d** Correlation analysis (Pearson, confidence interval = 95%) between the average cell sizes calculated via the TF and CML methods (n = 344). The boxplots represent the median and the 25th and 75th quartiles; the whiskers represent the minimum and maximum. The statistic method used was Student’s t test; **** represents p value < 0.0001, and “ns” represents “not significant”

We carefully manually labeled 362 seed coat cells as the gold standard and calculated the cell parameters. To evaluate the reliability of the CF and CML methods, we applied both methods to the manually labeled cells to obtain their areas. The cell areas calculated via the CML method showed no difference compared to those calculated manually, while the cell areas acquired via the CF method were significantly smaller than those acquired using the CML and manual methods (Fig. 6a, Additional file 4: Dataset S3). Both methods rely on labeled images, but the CF method generates 5–12% smaller data values than the CML method. This supports the idea that the CF method is biased because of the gaps between adjacent cells. The cell areas calculated via the CML and manual methods exhibit a significant positive correlation (Pearson’s R = 0.94) (Fig. 6b). These results reveal that the CML method is reasonably accurate and can achieve human-level performance.

To investigate the robustness of these methods, we applied them to 172 randomly selected seeds and obtained 344 images of seed coat cells (two images per seed). After segmentation with TWS or Cellpose, we created macros to implement the following steps automatically in Fiji. Finally, the cell parameters corresponding to all images were successfully and precisely quantified and exported (Additional file 4: Dataset S4). Based on the seed area, we calculated the seed surface area by considering the seed shape to be a standard sphere. By dividing the seed surface area by the average cell size, we then estimated the number of seed coat cells for each seed. Interestingly, we found that the CML method was highly precise and barely needed manual correction (negligible, if any), indicating excellent potential for high-throughput analysis, whereas the TF method consistently needed calibration.

Finally, the seed parameters, including the seed area, average cell size and number of cells, were quantified and visualized (Fig. 6c, Additional file 4: Dataset S1). The average values of cell size derived from the TF and CML method were 276.77 μm^2^ and 283.02 μm^2^, respectively, while the corresponding numbers of cells were 34,985.1 and 34,272.4. Accordingly, the average cell size and cell number calculated via the TF method were not significantly different from those calculate via the CML method (Fig. 6c). The data acquired via the TF and CML methods revealed an extremely strong positive correlation (Pearson’s R = 0.98), demonstrating the robustness and precision of these methods (Fig. 6d). These results indicate that our strategy is highly efficient for high-throughput seed coat cell quantification.

In summary, the CML method is highly accurate at both the individual cell level and the sample level. Additionally, the CML method eliminates the need for most manual corrections. Therefore, we chose to focus on the CML method for further analysis.

### Seed coat cell size measurement of developing seeds

**Fig. 7:**
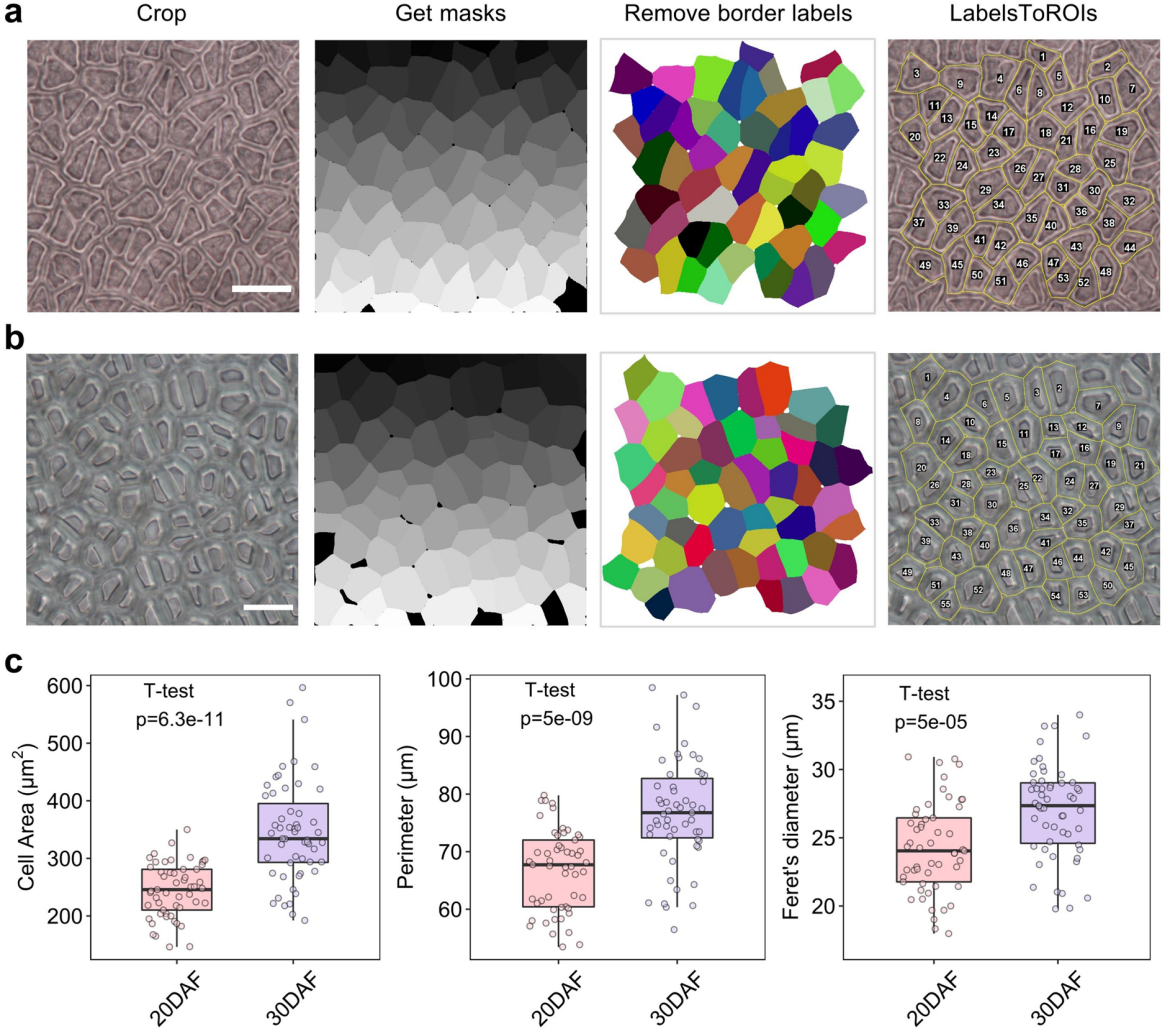
Cell size measurement of developing seed coats via the CML method. **a** Image processing procedure for 20 DAF seed coats. Bar = 30 μm. **b** Image processing procedure for 30 DAF seed coats. Bar = 30 μm. **c** Cell parameter comparison between 20 and 30 DAF seed coats. The boxplots represent the median and the 25th and 75th quartiles; the whiskers represent the minimum and maximum (n = 53, 55). The cropped and mask images for **a** and **b** are provided in Additional file 9, 10, 11, 12

Next, we investigated whether the CML method could be applied to the seed coat cells of developing seeds. We sampled developing seeds/ovules from siliques at 20 and 30 days after flowering (DAF) and fixed them with FAA solution. Then, the samples were rendered transparent in clearing solution. Subsequently, the seed coats were cut into small pieces and photographed under a differential interference contrast (DIC) microscope. Three cell layers of the developing seed coat could be observed, i.e., the epidermis, sub-epidermis, and palisade. The palisade layer was found to be the most characteristic and was used for further analysis (Additional file 9 and 11). Compared to those at 20 DAF, the seed coat cell walls at 30 DAF were thickened and became visible under bright-field imaging (Fig. 7a, b). We applied the CML method to these images (Fig. 7a, b) and achieved high performance in cell segmentation. Fifty-three and fifty-five intact cells were detected for 20 DAF and 30 DAF images, respectively, with corresponding average cell areas of 244.53 μm^2^ and 345.89 μm^2^. Consistent with our expectations, the cell size at 30 DAF was significantly increased compared to that at 20 DAF (Fig. 7c, Additional file 4: Dataset S5), revealing cell expansion during seed development. These results suggest that the CML method is reliable and applicable for cell quantification of developing seed coats.

### Quantitative evaluations of the embryo cell size of mature seeds

**Fig. 8:**
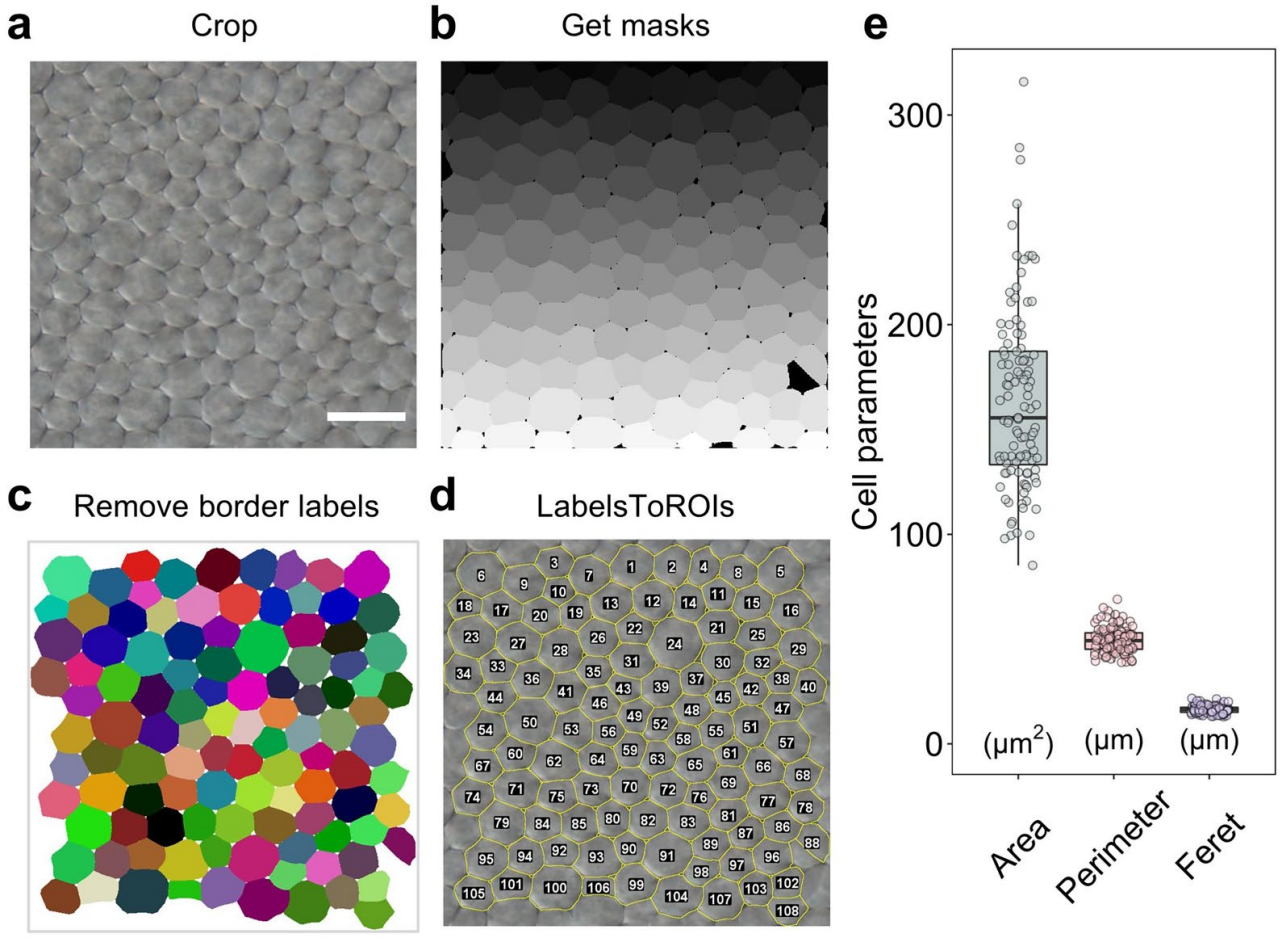
Embryo cell size measurement of mature seeds via the CML method. **a** A raw cell image was cropped. Bar = 30 μm. **b** The cell mask was acquired using Cellpose. **c** The border labels were removed by the MorphoLibJ plugin. **d** The label information was transformed into ROIs by the LabelsToROIs plugin. **e** The cell parameters were acquired from image (**a**). The boxplots represent the median and the 25th and 75th quartiles; the whiskers represent the minimum and maximum (n = 108). Images **a** and **b** are provided in Additional file 13 and 14

In addition to the seed coat, the embryo size also influences the seed size, thus arousing our interest in determining the embryonic cell area. We placed a mature seed in distilled water to soak and peeled off the seed coat, and then treated the embryo with clearing solution to make it transparent. The cells on the adaxial side of the outer cotyledon were photographed under a microscope (Additional file 13). The embryo cell shape was nearly circular and could be precisely segmented by Cellpose. We implemented the CML method on an embryo cell image and identified 108 intact cells with an average area of 164.13 μm (Fig. 8a–d). Other cell parameters were also generated (Fig. 8e, Additional file 4: Dataset S6). These results indicate that the application of the CML method can be extended to mature embryo cells in rapeseed.

### Silique wall cell size quantification

**Fig. 9:**
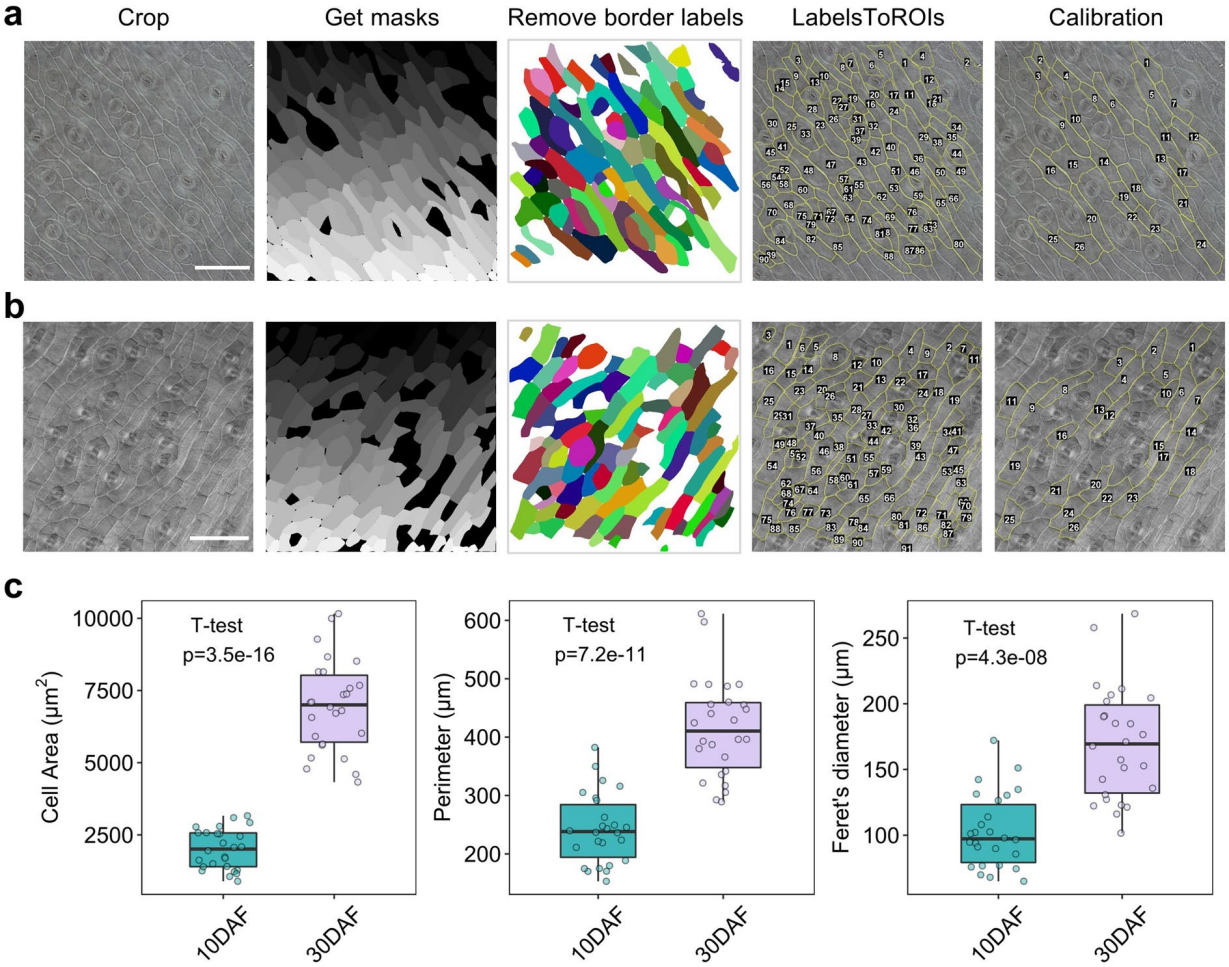
Cell size measurement of developing silique walls via the CML method. **a** Image processing procedure for 10 DAF silique walls. Bar = 100 μm. **b** Image processing procedure for 30 DAF silique walls. Bar = 200 μm.** c** Cell parameter comparison between the 10 DAF and 30 DAF silique walls. The boxplots represent the median and the 25th and 75th quartiles; the whiskers represent the minimum and maximum (n = 26, 26). The cropped and mask images for **a** and **b** are provided in Additional file 15, 16, 17, 18

The rapeseed pericarp/silique wall is composed of three layers, namely, the exocarp, mesocarp and endocarp [26]. The exocarp contains stomata cells and adjacent cells, which are irregular in shape and challenging to manually annotate. In previous studies, the silique cell parameters have mainly been calculated manually, which is labor intensive and arbitrary [8–10]. To test the feasibility of segmenting pericarp cells with Cellpose, we applied the CML method to silique wall samples. Developing siliques (10 DAF) and full-length siliques (30 DAF) were cut into small pieces and fixed with FAA solution. After clearing, the mesocarp and endocarp were carefully scraped off. The outer surface of the exocarp was photomicrographed (Additional file 15, 17) and processed with the CML method (Fig. 9a, b). In total, 90 cells were successfully characterized in images of 10 DAF samples. Nonetheless, a fair number of cell outlines were distributed on stomata and adjacent cells, meaning that they were not applicable for quantifying silique wall cells. In addition, a few cells were over-split. We deleted the ROIs corresponding to falsely detected cells and merged the ROIs of over-split cells in the “ROI manager” window and eventually obtained 26 ROIs. The remaining ROIs were reimported into the ROI manager and measured, and then the results were generated in the “Results” window. The average area of 10 DAF silique wall cells was 2000.22 μm^2^. We processed the 30 DAF samples and manually corrected them in the same way as the 10 DAF samples. Twenty-six correctly segmented cells remained from the 91 originally detected cells, with an average area of 6971.57 μm^2^. Despite the need for considerable calibration, we believe that the CML method is still more efficient and accurate for this task than the manual method. In fact, the calibration work could be reduced by means of appropriate size filtering in MorphoLibJ. We compared the cell area, perimeter and Feret’s diameter of the 30 DAF silique samples to those of the 10 DAF samples and found a dramatic increase in all three parameters (Fig. 9c, Additional file 4: Dataset S7). This is consistent with the enlargement of siliques during their development. These results demonstrate that the wild range of applications of the CML method includes the measurement of rapeseed silique wall cells.

## Discussion

Here we have introduced a low-cost, high-efficiency and high-throughput method for detecting and segmenting various cells from different organs in rapeseed. We simplified the typical photomicrograph procedure by skipping the fixation step and acquired high-quality images for cell quantification (Fig. 1). Three methods were developed for processing the cell images, either manually or automatically. We first developed the TF method by classifying cell cavities/walls with TWS and correctly detected most seed coat cells (Fig. 3). However, the TF method still suffers from a need for manual calibration. The CF method, in which we segment cells with Cellpose and automatically quantify them with Fiji, achieved good performance but exhibited systematic error. To circumvent this error and the need for manual calibration, we developed the CML method, in which the border labels are removed by MorphoLibJ and ROIs are detected by LabelsToROIs. The CML method was found to achieve human-level performance on bright-field images of mature/developing seed coat cells and mature embryo cells (Figs. 5, 7 and 8) and to exhibit acceptable performance on images of silique wall cells (Fig. 9), revealing a high tolerance to miscellaneous cell types and shapes. Through the large-scale quantification of 362 manually labeled seed coat cells and 344 seed coat images from 172 seeds, we demonstrated the robustness and accuracy of the CML method at both the individual cell level and the sample level (Fig. 6). Currently, the study of plant functional genomics is accelerating due to the availability of multiomics data and high-throughput phenotyping methods [27]. We anticipate that our strategy based on Cellpose will contribute to the data processing of plant microphenotypes and the development of plant phenomics.

Cellpose is a powerful tool for segmenting cells of highly diverse shapes with great precision [21]. Cellpose generates results in the form of labeled images. For users without programming skills, there is still a gap between such labeled images and the final readable results. Here, we have provided a step-by-step procedure for transforming the information stored in label images into cellular properties, thus partially filling this gap. We have also created several macros for batch analysis. We exclude the cells on the edges by means of MorphoLibJ because they are not intact, and thus, their inclusion would detrimentally affect the average cell parameters. The Fiji plugin LabelsToROIs can convert label images into ROIs in a user-friendly manner [23]. Thus, by integrating Cellpose, MorphoLibJ and LabelsToROIs, we have created the CML method, which can function accurately and automatically. Our strategy is flexible and can be applied in a broad range of scenarios. Importantly, any kind of labeled images can be fed into this method with minor modifications, and thus, its use is not limited to rapeseed cells.

We focused on the palisade layer of the seed coat when we acquired the cell images. The mature seed coat consists of three layers. The palisade layer is the main protective tissue that constrains the seed size and thus attracts our primary interest. The epidermis/sub-epidermis layers are flattened or crushed at maturity [11], which are hard to visualize and therefore cannot be quantified using this method. The aleurone layer is a single layer of live cells with a rectangular shape, which could theoretically be measured using this method. Nevertheless, the aleurone layer is not taken into consideration here because it does not constrain the seed size.

The cells of palisade layer of mature seed coat are dead with their rigid cell walls remain [11, 25]; therefore, it is reasonable to postulate that the cell shape and size are not affected by water absorption or treatment with clearing solution. However, the cells of developing seeds are alive, and their shape and size may be altered if not fixed. Thus, the fixation step can be skipped for mature seed coats but not for developing tissues.

We have identified several factors that may lead to moderate discrepancies in practice. First, the image quality significantly affects the accuracy of cell segmentation. The clarity and sharpness of the cell margins exert a strong influence on the segmentation results. Second, the number of cells in the seed coat may be miscalculated considering that (i) some seed shapes are different from a standard sphere and (ii) the cells display strong heterogeneity. According to our observation, the average difference between cell sizes from opposite regions of the seed sphere is 6.274% (Additional file 4: Dataset S4), which is relatively small; therefore, we believe that these regions can be representative for the major part of the whole seed. Although the estimate of the number of seed coat cells may be rough, it provides us with useful information about seed coat cell proliferation. Third, some seed coat cells from developing seeds are in the process of dividing in a complex and asymmetric way, and such cells tend to be under-segmented by Cellpose (multiple cells are merged into one) (Fig. 7a). These discrepancies are quantitatively negligible and can be identified and corrected easily when we add the ROIs to the original images.

The integrated advanced deep learning-based algorithm, Cellpose, has demonstrated its ability to segment cells from highly varied organisms or tissues [21]. In particular, Cellpose works well for the rectangle-like or polygonal cells of the rapeseed silique wall. However, the stomata and adjacent cells are often incorrectly segmented, necessitating further manual calibration. Cellpose achieves promising performance for regular-shaped or polygon-like cells, but not for puzzle-shaped cells. Recently, LeafNet, a tool that can localize stomata and segment pavement cells, was released for processing bright-field microscopy images of leaf epidermis [28]. We have made substantial efforts to segment silique wall cells with LeafNet but have rarely obtained acceptable results. This may partially be attributed to the effects of cell shape diversity and image quality. Nevertheless, the strategy of identifying the stomata first and segmenting other cells on the stomata-masked images appears promising. We envision that more sophisticated tools will be developed to solve this problem.

Another software commonly used for the measurement of cell parameters in plants, MorphoGraphX, is designed to reconstruct 3D images from datasets obtained via confocal imaging and performs well on highly curved organs [29]. However, obtaining high-quality images can be expensive and challenging. In contrast to MorphoGraphX, our method can only be applied to 2D images acquired from planar tissues; however, this method is easy to implement and does not require costly equipment, making it a useful complement to MorphoGraphX. Importantly, our method provides several macros that run automatically, which is very attractive for high-throughput analysis.

## Conclusion

By combining simplified image preparation, reliable cell segmentation and automated cell quantification, we have developed three methods to acquire the cell parameters of the seed coat, embryo and silique wall in rapeseed. Among these three methods, the CML method achieves the best performance at both the individual cell level and the sample level. The proposed procedure can be applied for the low-cost, high-throughput quantitative evaluation of diverse cell types, facilitating studies of rapeseed and other crops in the fields of developmental biology, functional genomics and microphenomics.

## Methods

### Reagents

1. Chloral hydrate (Rhawn, CAS No: 302-17-0)
2. Glycerol (Sinopharm Chemical Reagent, CAS No: 5-81-5)
3. Ethanol (Sinopharm Chemical Reagent, CAS No: 6-17-5)
4. Acetic acid (Sinopharm Chemical Reagent, CAS No: 6-19-7)
5. Formaldehyde (Sinopharm Chemical Reagent, CAS No: 5-00-0).

### Equipment

1. Glass slide (Sail Brand, CAS No: 7101)
2. Microscope cover glass (Citotest Scientific, CAS No: 10211818C)
3. Tweezer (Vetus, model: ST-10)
4. Surgical blade (Jinhuan Medical, model: K3-24)
5. Stereo microscope (Olympus, SZX16)
6. Differential interference contrast (DIC) microscope (Nikon, ECLIPSE 80i) with CCD camera (Nikon, DS-Ri1).

### Software

- 1. Fiji (https://imagej.net/software/fiji/?Downloads)
- 2. Java version 8 or above (https://www.java.com/en/download/)
- 3. Trainable Weka Segmentation (https://imagej.net/plugins/tws/)
- 4. Cellpose (https://www.cellpose.org/)
- 5. Anaconda (https://www.anaconda.com/products/individual).

### Plant materials

The mature dry seeds of rapeseed breeding material 7–5 were used for image acquisition and cell parameter quantification. The developing seeds were acquired from ZY50 at 20 and 30 days after flowering. The developing siliques were acquired from ZY50 at 10 and 30 days after flowering.

### Images acquisition of mature seeds and seed coat cells

1. Choose fully grown seeds of rapeseed and photograph each seed under a stereomicroscope.
2. Put individual seed in a 2 mL centrifuge tube, and submerge the seed in 200 μL distilled water for 6 h or overnight.
3. Cut the seeds in half and peel off the seed coat, then submerge the seed coat in 100 μL clearing solution (Hoyer’s solution, chloral hydrate:water:glycerol = 8 weight:3 volume:1 volume) for 1 day.
4. Put the seed coat on a glass slide with a tweezer, and cut a small piece in the middle of each half of the seed coat (the equatorial cross-sectional area) in order to flatten it (the size is about 1 mm × 1 mm).
  - Make sure the outer surface of the seed coat is upward, so that the cell images will be clear and sharp.
  - The cells of the hilar region are highly compact; therefore, this region should be avoided.
5. Add a few drops of clearing solution and cover the sample with a microscope cover glass carefully.
6. Capture images of the seed coat cells under a 400 × optical microscope, make sure the edges of the cells are clear; for a batch of samples, use the same brightness and exposure time to make the images uniform. Raw images with resolution 96–300 dpi (dot per inch) are suitable for further analysis. This resolution requirement is applicable to all other raw images in this article.

### Seed coat cell images acquisition of developing seeds

1. Choose developing siliques of rapeseed and peel off the silique wall, then submerge the developing seeds/ovules in FAA solution (ethanol:water:acetic acid:formaldehyde = 50:40:5:5, in volume) for 1 day. Note that the seeds after 30 DAF have thickened coat cell walls which prevent external solution to immerse, therefore, these seeds need to be cut in half first.
2. Wash the seeds two times with 75% ethanol and soak them in 200 μL clearing solution for 2 days.
3. Put the seed on a glass slide and cut into small pieces (the size is about 2 mm × 2 mm). Squeeze out the contents of seed cavity carefully. Make sure the outer surface of the seed coat is upward and the hilar region is avoided. For some materials (such as rapeseed variety Westar), the epidermis is thick and need to be cut and scraped to expose the palisade layer.
4. Add a few drops of clearing solution and cover the sample with a microscope cover glass.
5. Capture images of the seed coat cells under a 400 × optical microscope. To acquire clear images, we use differential interference contrast (DIC) model to deal with multiple cell layers.

### Embryo cell images acquisition of mature seeds

1. Let the mature seed imbibe for 6 h.
2. Peel off the seed coat, then submerge the embryo in 200 μL clearing solution for 1 day.
3. Put the embryo on a glass slide, push the outer cotyledon aside and cut into half. Make the adaxial side upward and add a few drops of clearing solution, then cover the sample with a cover glass.
4. Capture images of the embryo cells under a 400 × optical microscope. Use DIC model to obtain clear images.

### Silique wall cell images acquisition of developing siliques

1. Crosscut several pieces of developing silique wall of rapeseed (about 1 cm long), then submerge the samples in FAA solution for 1 day.
2. Wash the samples twice with 75% ethanol and soak them in clearing solution for 2 days.
3. Put the sample on a glass slide and scrape off the mesocarp and endocarp carefully. Make the outer surface of the exocarp upward.
4. Add a few drops of clearing solution and cover the sample with a cover glass.
5. Capture images of the silique wall cells under a 200 × (for 10 DAF samples) or 100 × (for 30 DAF samples) optical microscope. Use DIC model to acquire clear images.

### Seed size measurement using Fiji

1. Analysis procedure for a single image.
  - Open an image of the seed with Fiji and set a global scale (Analyze > Set Scale).
  - Convert the image to 8-bit greyscale (Image > Type > 8-bit).
  - Set threshold, for instance, to “0–100” (Image > Adjust > Threshold).
  - Measure seed area (Analyze > Analyze Particles), set “Size” to “1-infinity” (after Scale set), select “Clear results”, “Include holes”, “Summarize”, “Add to manage” and “In situ show”, then press “OK”.
  - Re-open the seed image and add ROIs to the original image (Image > Overlay > From ROI Manager), then save the image to a new folder (File > Save As > Tiff).
  - Export data to.csv file from the “Summarize” window (File > Save As).
2. Running a macro for a batch of images

All the processing steps can be recorded in Fiji (Plugins > Macros > Record). With the help of the “Recorder”, we can create a macro to automatically analysis of the seed images. For example, we create a macro named “seedsize.ijm”, with the script provided in Additional file 3. Users can run the macro file by going to “Plugins > Macros > Run”, or just drag the file to Fiji window and click “Run”, then a batch of files in a single folder will be processed automatically. Also, users can modify the parameters and create their own macros. After the running, all data of the images can be exported to.csv file from the “Summarize” window (File > Save As).

### Cell size measurement using Trainable Weka Segmentation and Fiji software

We name this method the TF method.

1. Preparing cell images
  - Open an image of the seed coat cells with Fiji and set a global scale (Analyze > Set Scale).
  - Make a rectangular selection around the image including about 100 cells, then go to “Image > Crop”; Mind that the scale bar should be excluded.
  - For a set of images with the same magnification, the crop command can be recorded and run as a macro (Process > Batch > Macro); For instance, copy and paste the following text into the “Batch Process” window, then choose the “Input” and “Output” folder and click “Process”.
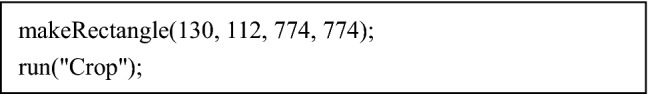
2. Cell segmentation using Trainable Weka Segmentation
  - Open a cropped image with Fiji, then go to “Plugins > Segmentation > Trainable Weka Segmentation”.
  - Click “Settings” to change the class names, click “Create new class” to increase the number of classes.
  - Make a freehand selection around the seed cavity regions and click “Add to class 1”, select the seed wall regions and click “Add to class 2”, then click “Train classifier”; mind that at least two selections are needed for the training.
  - The segmentation result will be overlaid with the corresponding class colors. If the result is not satisfied, select the misclassified regions to the right class, click “Train classifier” another time.
  - For a single image, click “Get probability” and save the image to a new folder. For a batch of images, click “Apply classifier”, choose all the images, then a dialog will pop up ask whether to store on the disk, click “Yes”; another dialog will pop up to ask whether to create probability maps, click “Yes”. Then the plugin will perform the image segmentation based on the current classifier and store the probability maps in the select folder.
  - For more information about TWS plugin, see https://imagej.net/plugins/tws/.
3. Cell count for a single image
  - Open the probability map, go to “Image > Color > Make Composite”, choose “Grayscale”, then click “OK”.
  - Two windows will pop up, close “C2-Probability maps” window, keep “C1-Probability maps” window in front.
  - Set threshold (Image > Adjust > Threshold), select “Black Background”, click “Apply > Convert to Mask”.
  - Process > Binary > Fill holes.
  - Analyze > Analyze Particles, set “Size” to “30–400” (after Scale set), select “Clear results”, “Include holes”, “Summarize”, “Add to manage” and “In situ show”, then press “OK”.
  - Re-open the original image and add ROIs (Image > Overlay > From ROI Manager), save the image to a new folder.
  - Export data to. csv file from the “Summarize” window.
4. Cell count for a batch of images

We also create a macro named “TWS_cell_count.ijm” that can process the probability maps and count cells automatically. The script is provided in Additional file 3.

5. Cell size calculation

Open the saved images with overlays, then calibrate the selections and acquire the accurate number of cells in this image. If over half of the cells are within the cropped area, count them in; otherwise ignore them. Calculate the cell size according to the following formula.

$$Average \,cell\, size=\frac{cropped\, area ({\mathrm{\mu m}}^{2}) }{cell\, number}$$

For example, if there are 97 cells in a 170.86 × 170.86 μm^2^ area, then the average cell size is 170.86 × 170.86/97 = 300.96 μm^2^.

### Cell size measurement using Cellpose and Fiji

We name this method the CF method.

1. Preparing cell images.
  The “Set Scales” and “Crop” steps are the same as the TF method.
2. Get cell masks with Cellpose.
  - Open Anaconda Navigator, click “Environment > Create”, enter a name and choose “Python 3.8.12”, to create a Python3.8 environment for Cellpose.
  - At “Home” interface, choose the new-created Python3.8 environment and launch “Spyder”.
  - Type in “pip install Cellpose” to install Cellpose package, and install dependent packages if missing. For more information, see Cellpose document (https://cellpose.readthedocs.io/en/latest/).
  - Open file “getcellmask.py” and change the input and output folder, then run the script. The script is provided in Additional file 3. The cell masks will be saved in the output folder. The cell mask files can be opened by Fiji/ImageJ software to view the segmented cells.
3. Cell size measurement for a single image.
  - Open an image of the cell masks with Fiji and set a scale (Analyze > Set Scale).
  - Process > Find Edges.
  - Set threshold to “0–0” (Image > Adjust > Threshold).
  - Analyze > Analyze Particles, set “Size” to “70–650”, set “Circularity” to “0.4–1”, select “Display results”, “Exclude on edges”, “Clear results”, “Include holes”, “Summarize”, “Add to manage” and “In situ show”, then press “OK”.
  - Re-open the original image and add ROIs (Image > Overlay > From ROI Manager), save the image to a new folder.
  - Export average cell size data from the “Summarize” window. Export single cell size data from the “Results” window.
4. Cell size measurement for a batch of images.

We create a macro named “celp_mask_sum.ijm” that can process the mask images and calculate the cell sizes automatically. The script is provided in Additional file 3.

### Cell size measurement using Cellpose, MorphoLibJ and LabelsToROIs

We name this method the CML method.

1. Preparing cell images.
  The “Set Scales” and “Crop” steps are the same as the TF method.
2. Get cell masks with Cellpose.
  This step is the same as the CF method.
3. Cell size measurement for a single image.
  - Open an image of the cell masks with Fiji. Install MorphoLibJ and LabelsToROIs plugin if missing.
  - Plugins > MorphoLibJ > Binary Images > Connected Components Labeling, set “Connectivity” = 4 and “Type of result” = 16 bits.
  - Plugins > MorphoLibJ > Label Images > Label Size Filtering, set “Operation” = “Greater_Than” and “Size Limit” = 100, this will keep the labels that are greater than 100 pixels.
  - Plugins > MorphoLibJ > Label Images > Remove Border Labels, choose “Left”, “Right”, “Top” and “Bottom”, click “OK”. Then save this file.
  - LabelsToROIs > Single Image, browse the original image and label image, then click “Next”, a new window will pop up and all ROIs will show on the original image. Edit ROIs in “ROI Manager” window and click “Update ROIs”. Click “Save CSV Table” and the results will be saved to.csv file. Mind that the file path should be English characters.
4. Cell size measurement for a batch of images.

We create a macro named “celp_mask_LabelsToROIs.ijm” that can transform the mask images to LabelsToROIs format. Put LabelsToROIs format files and the original files in the same directory, then go to “LabelsToROIs > Multiple Images”, browse the path to the directory and click “Run”, LabelsToROIs will create a result file for each original image file. Run “csv_sum.R” by Rstudio in this directory and a summary file named “SummaryAll.csv” will be generated. All the scripts are provided in Additional file 3.

### Seed coat cell number calculation

The seed shape of rapeseed is nearly spherical; therefore, the seed surface area (4πR^2^) is four times of seed area (πR^2^). We can calculate the number of seed coat cells approximately as the seed surface area divided by the average cell size. The formula is as following.

$$seed \,coat \,cell\, number = \frac{seed \,surface \,area \left({\mathrm{mm}}^{2}\right)\times {10}^{6}}{average \,cell\, size ({\mathrm{\mu m}}^{2})}= \frac{seed \,area \left({\mathrm{mm}}^{2}\right)\times {10}^{6}\times 4 }{average \,cell\, size ({\mathrm{\mu m}}^{2})}$$

For instance, if the seed area is 2.6 mm^2^ and the average cell size is 251.9 μm^2^, then the seed coat cell number is 2.6 × 10^6^ × 4 / 251.9 = 41,286.

## Supplementary Information

**Additional file 1. **A seed image acquired under a stereomicroscope.

**Additional file 2. **A cell image of mature seed coat acquired under 400 × optical microscope.

**Additional file 3. **ImageJ macros used in this study.

**Additional file 4.** Raw data of all the figures.

**Additional file 5.** **Figure S1.** Detection of seed coat cells by the TF method.

**Additional file 6.** A cropped image of mature seed coat cells.

**Additional file 7.** **Figure S2**. Cell quantification of seed coat by the CF method.

**Additional file 8.** Cell masks of mature seed coat generated by Cellpose.

**Additional file 9.** A cell image of 20 DAF seed coat acquired under 400 × optical microscope.

**Additional file 10.** Cell masks of 20 DAF seed coat generated by Cellpose.

**Additional file 11.** A cell image of 30 DAF seed coat acquired under 400 × optical microscope.

**Additional file 12.** Cell masks of 30 DAF seed coat generated by Cellpose.

**Additional file 13.** A cell image of mature embyro acquired under 400 × optical microscope.

**Additional file 14.** Cell masks of mature embyro generated by Cellpose.

**Additional file 15.** A cell image of 10 DAF silique wall acquired under 200 × optical microscope.

**Additional file 16.** Cell masks of 10 DAF silique wall generated by Cellpose.

**Additional file 17.** A cell image of 30 DAF silique wall acquired under 100 × optical microscope.

**Additional file 18.** Cell masks of 30 DAF silique wall generated by Cellpose.

## Data Availability

All data are available in the main text or the Additional files.
