# Supplementary figures and images for: A simple and efficient method to quantify the cell parameters of the seed coat, embryo and silique wall in rapeseed
Source: Plant Methods. 2022 Nov 3;18:117. doi: 10.1186/s13007-022-00948-1 (PMC9632141; doi:10.1186/s13007-022-00948-1)

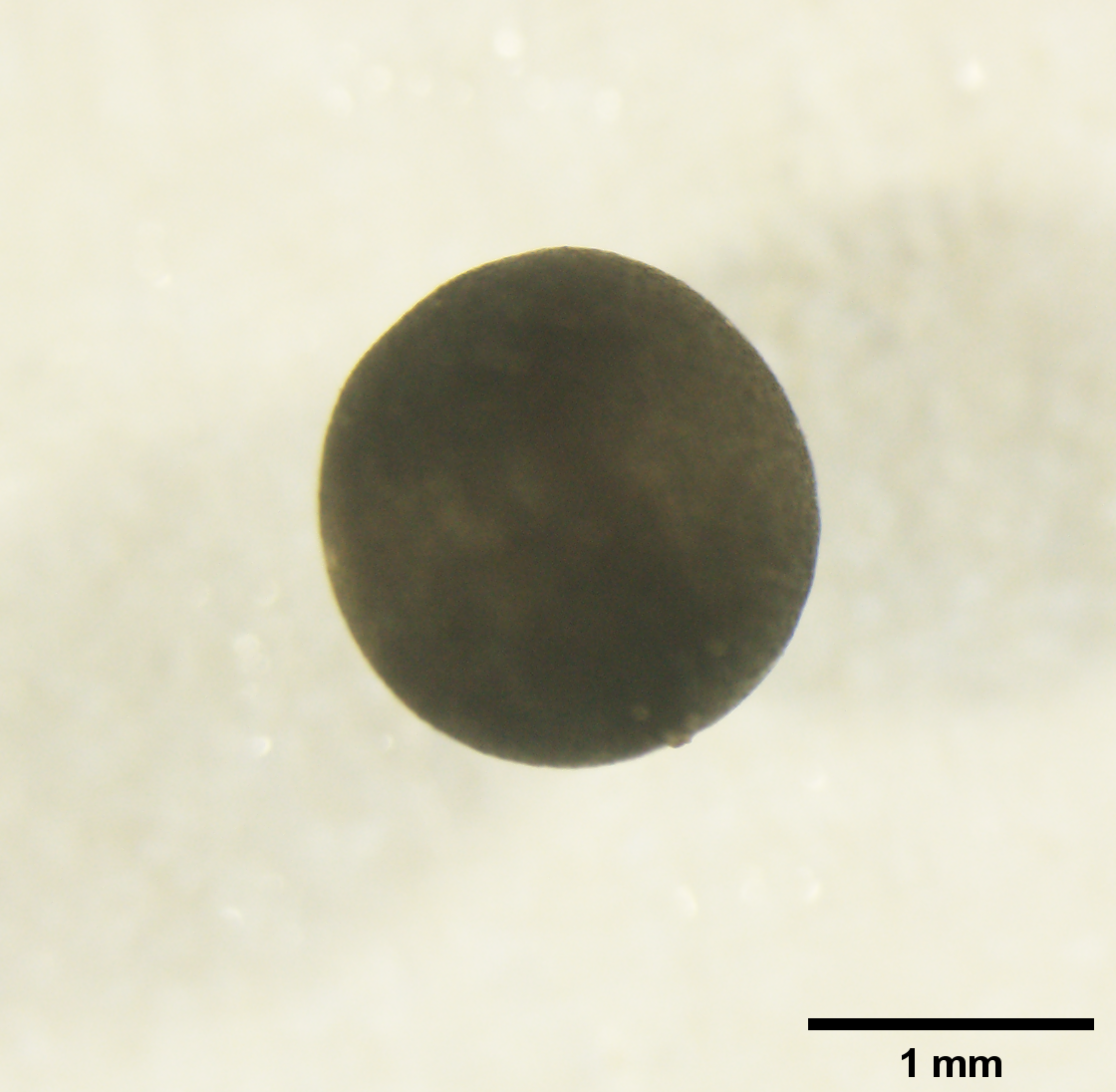

Supplement: Supplementary file 1 — Additional file 1. A seed image acquired under a stereomicroscope. [file 13007_2022_948_MOESM1_ESM.tif]

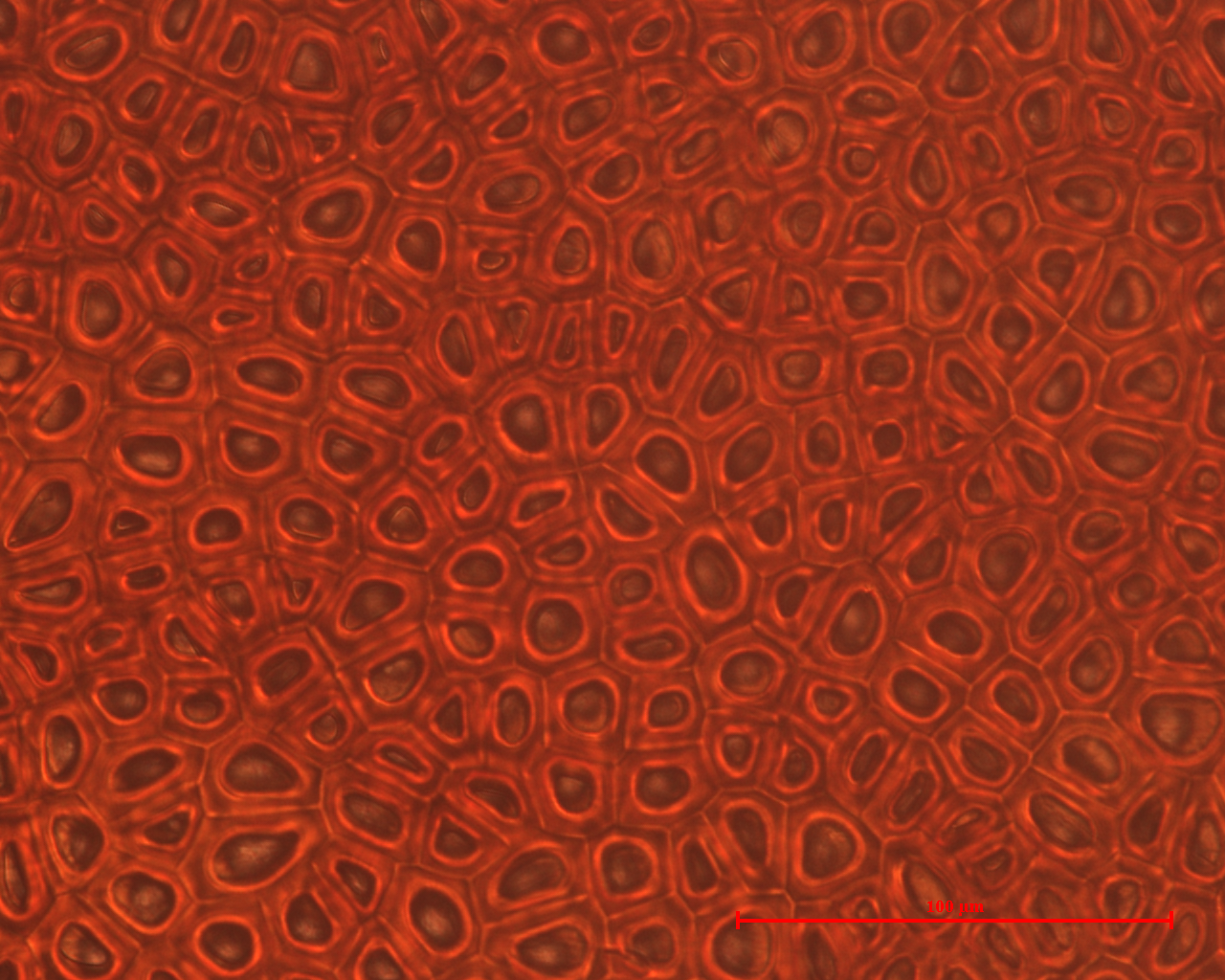

Supplement: Supplementary file 2 — Additional file 2. A cell image of mature seed coat acquired under 400 × optical microscope. [file 13007_2022_948_MOESM2_ESM.tif]

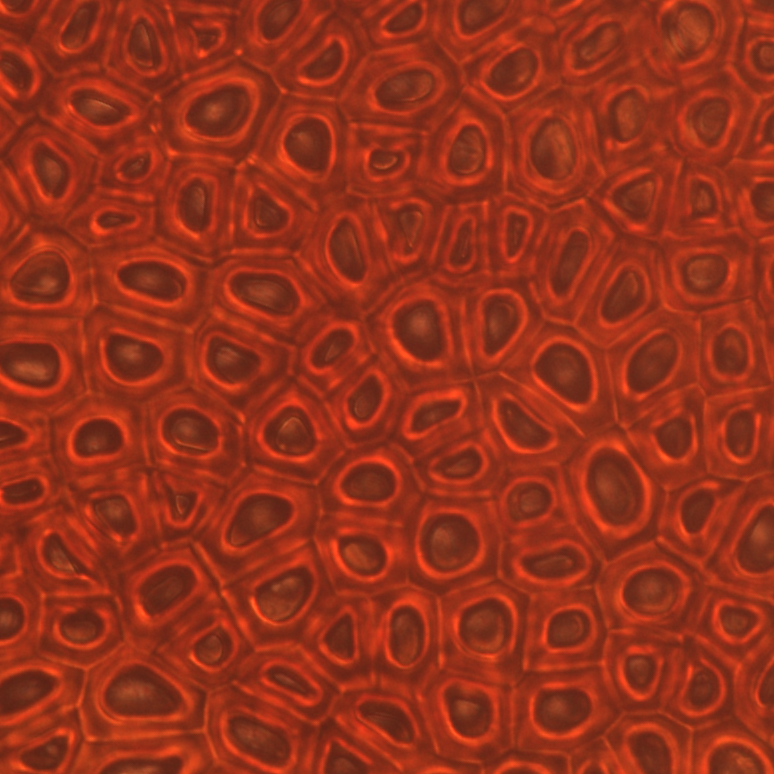

Supplement: Supplementary file 6 — Additional file 6. A cropped image of mature seed coat cells. [file 13007_2022_948_MOESM6_ESM.tif]

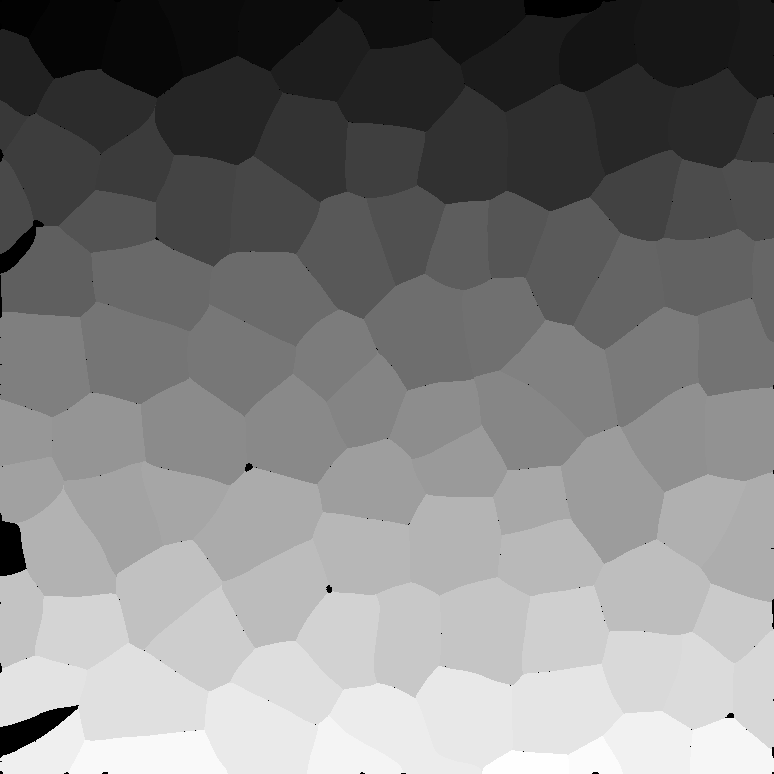

Supplement: Supplementary file 8 — Additional file 8. Cell masks of mature seed coat generated by Cellpose. [file 13007_2022_948_MOESM8_ESM.tif]

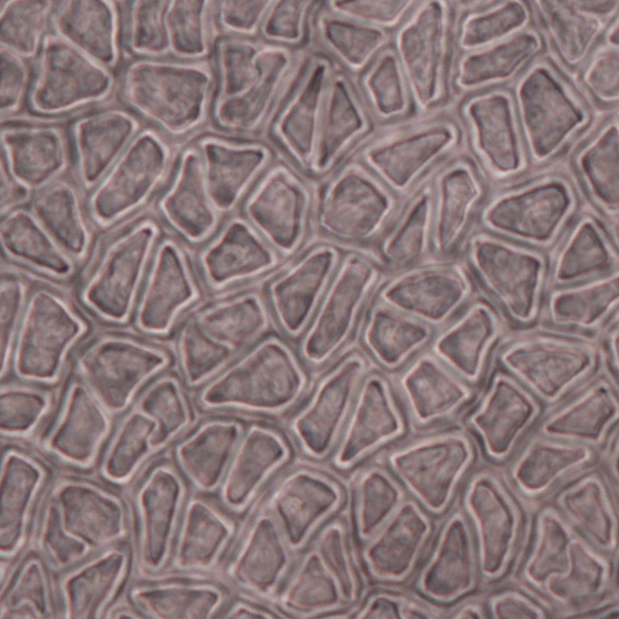

Supplement: Supplementary file 9 — Additional file 9. A cell image of 20 DAF seed coat acquired under 400 × optical microscope. [file 13007_2022_948_MOESM9_ESM.tif]

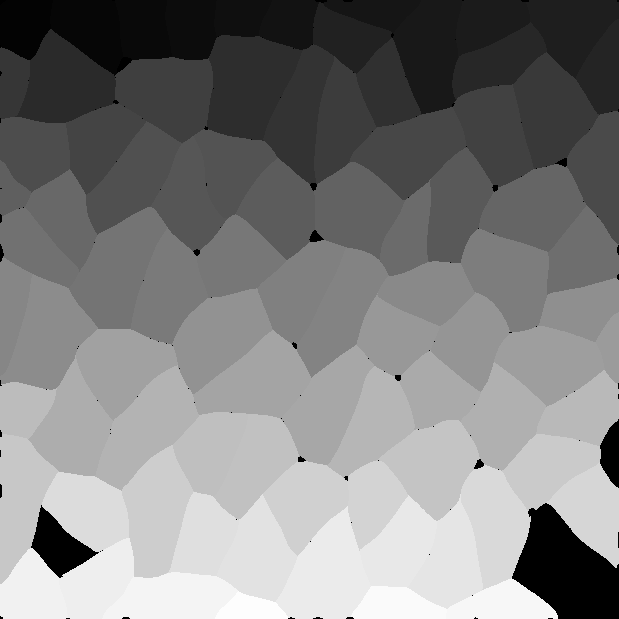

Supplement: Supplementary file 10 — Additional file 10. Cell masks of 20 DAF seed coat generated by Cellpose. [file 13007_2022_948_MOESM10_ESM.tif]

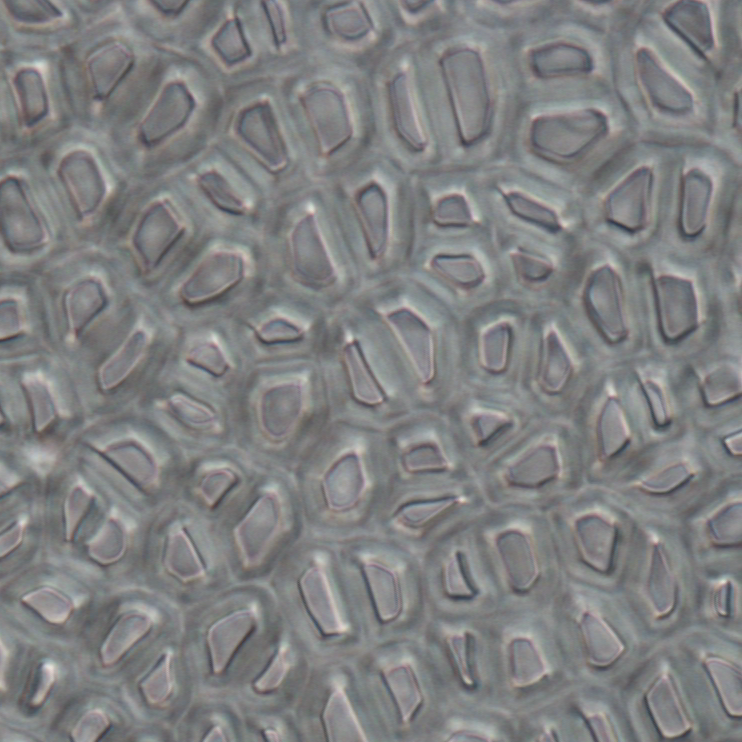

Supplement: Supplementary file 11 — Additional file 11. A cell image of 30 DAF seed coat acquired under 400 × optical microscope. [file 13007_2022_948_MOESM11_ESM.tif]

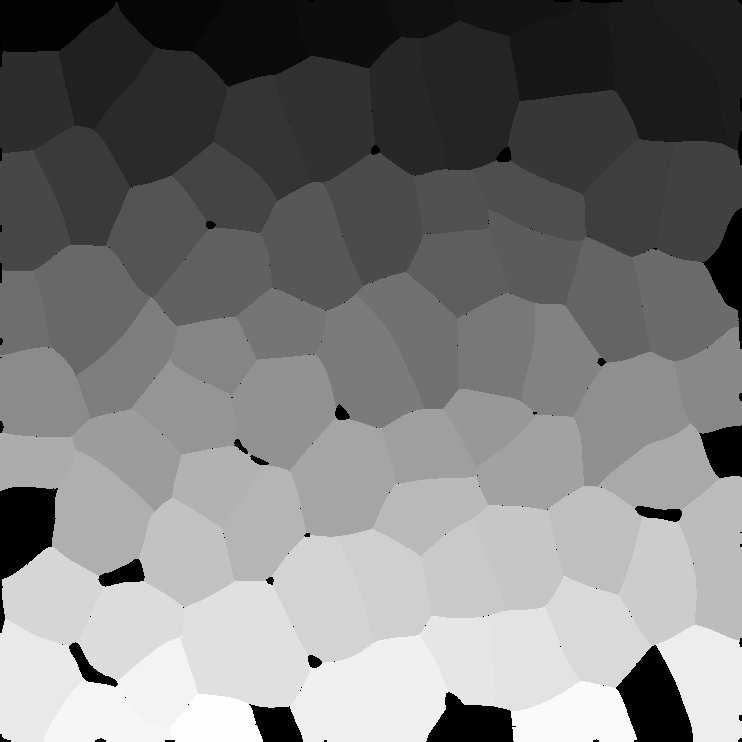

Supplement: Supplementary file 12 — Additional file 12. Cell masks of 30 DAF seed coat generated by Cellpose. [file 13007_2022_948_MOESM12_ESM.tif]

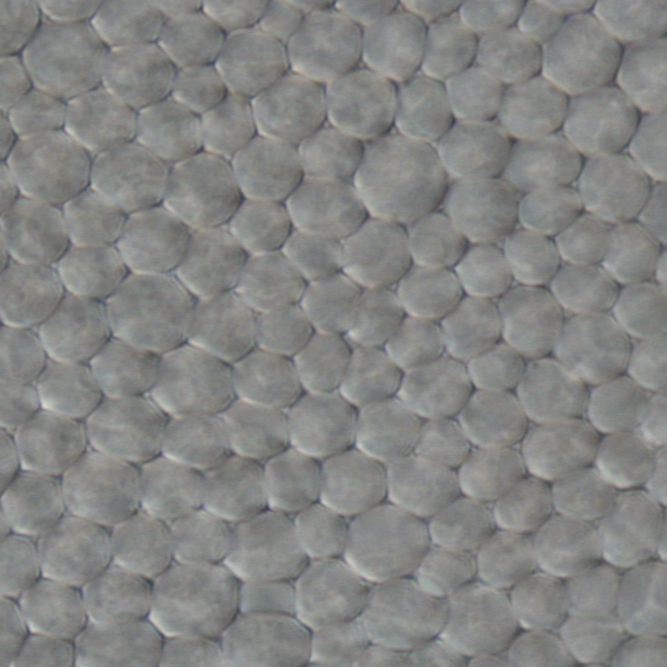

Supplement: Supplementary file 13 — Additional file 13. A cell image of mature embyro acquired under 400 × optical microscope. [file 13007_2022_948_MOESM13_ESM.tif]

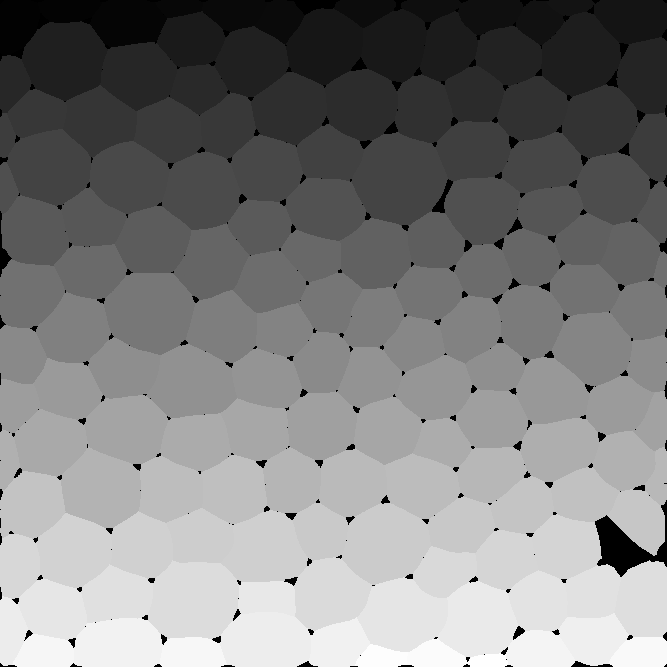

Supplement: Supplementary file 14 — Additional file 14. Cell masks of mature embyro generated by Cellpose. [file 13007_2022_948_MOESM14_ESM.tif]

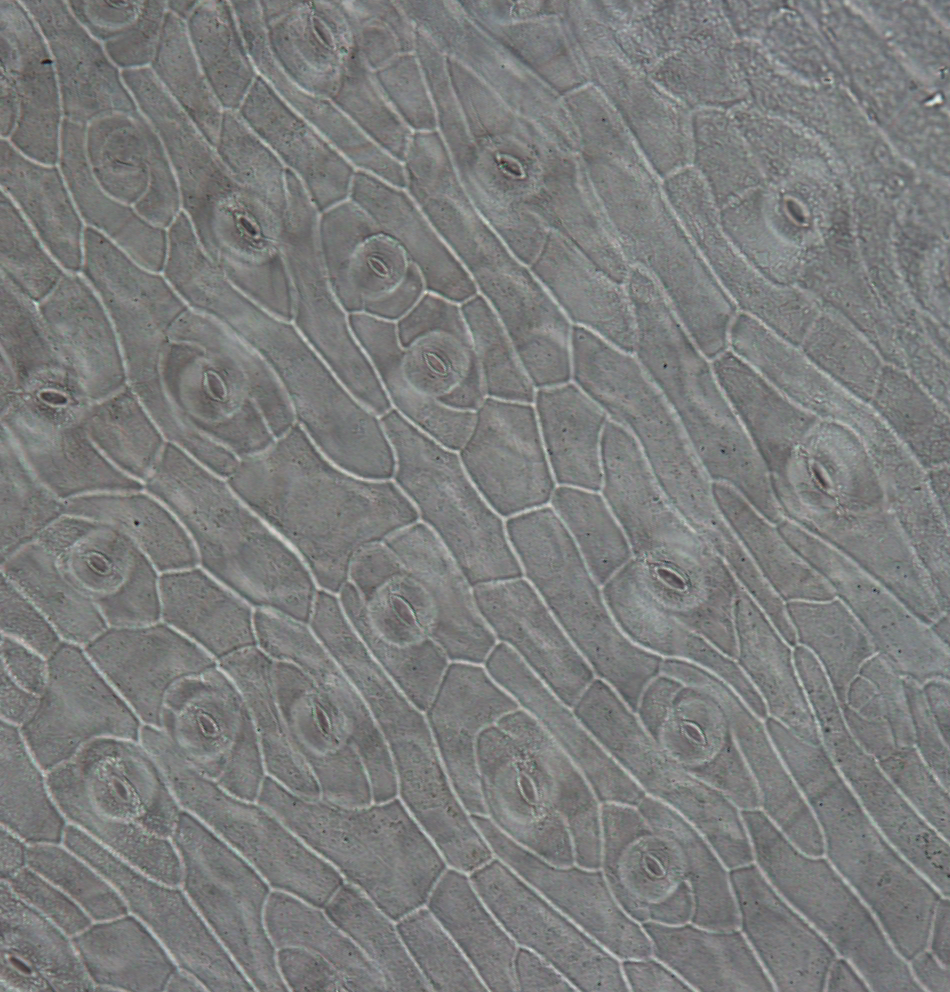

Supplement: Supplementary file 15 — Additional file 15. A cell image of 10 DAF silique wall acquired under 200 × optical microscope. [file 13007_2022_948_MOESM15_ESM.tif]

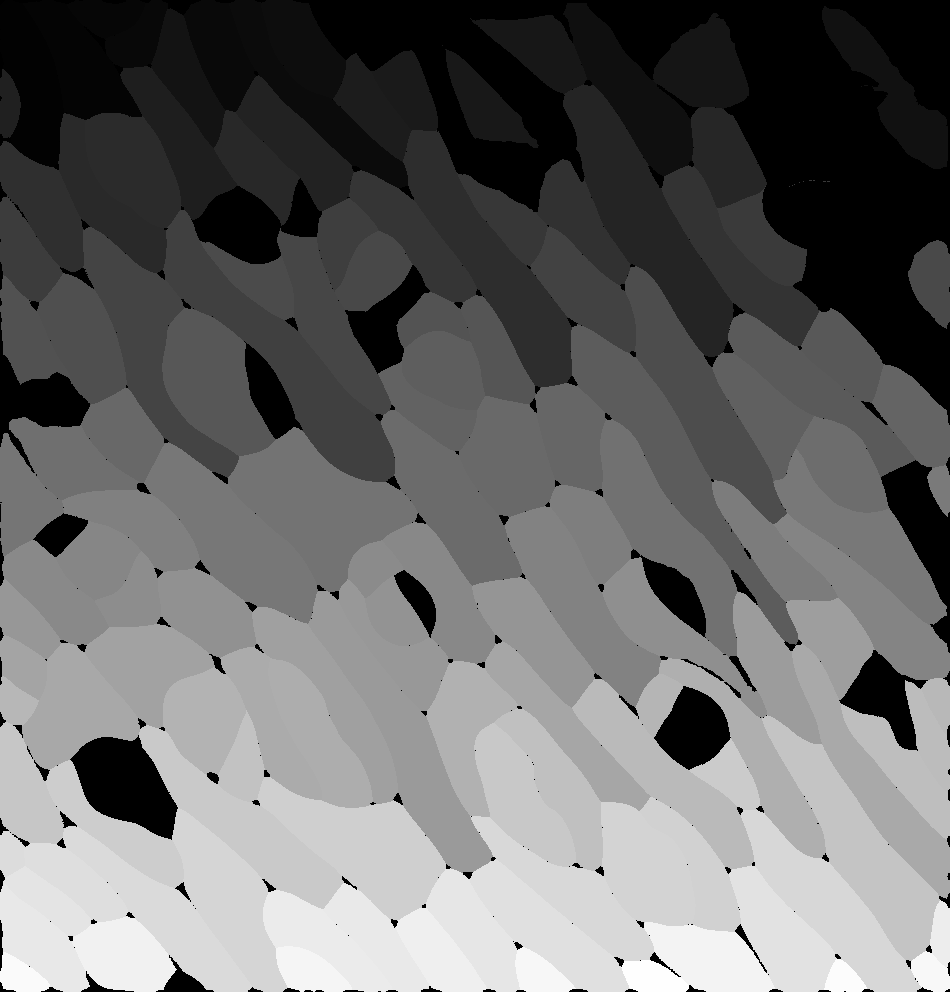

Supplement: Supplementary file 16 — Additional file 16. Cell masks of 10 DAF silique wall generated by Cellpose. [file 13007_2022_948_MOESM16_ESM.tif]

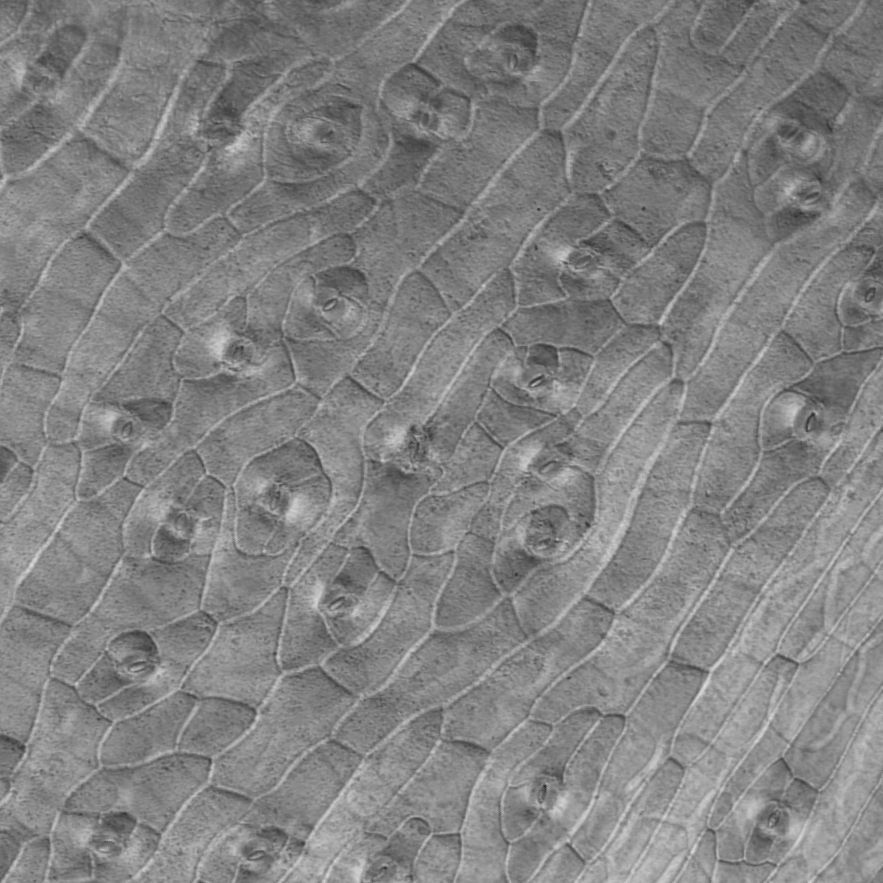

Supplement: Supplementary file 17 — Additional file 17. A cell image of 30 DAF silique wall acquired under 100 × optical microscope. [file 13007_2022_948_MOESM17_ESM.tif]

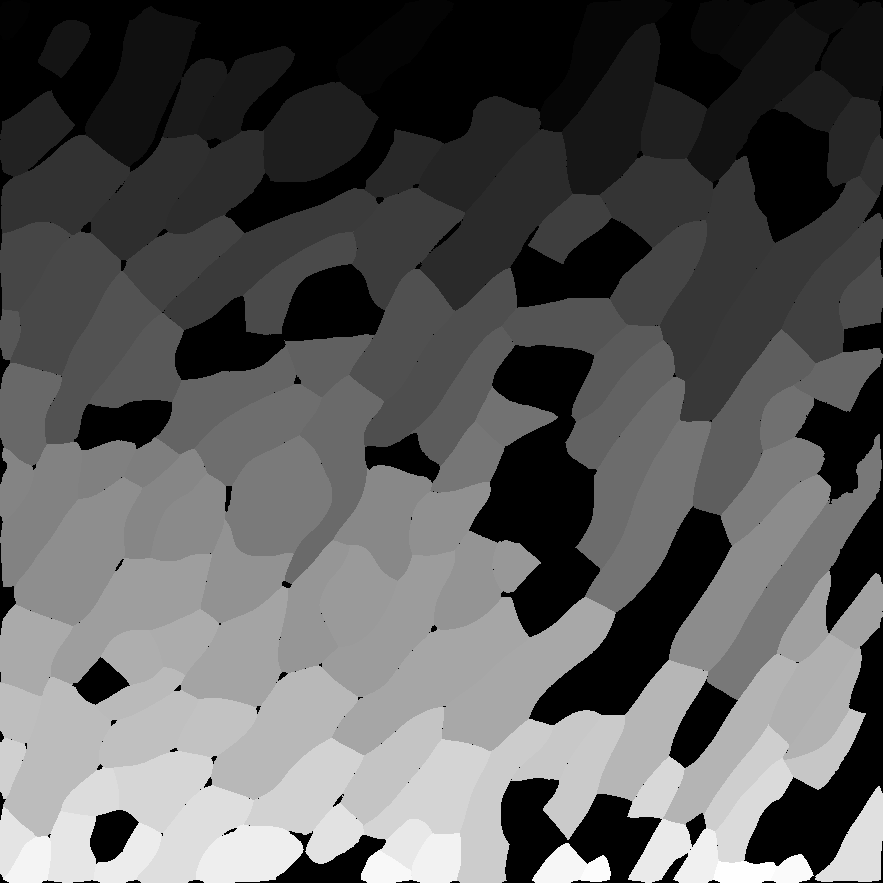

Supplement: Supplementary file 18 — Additional file 18. Cell masks of 30 DAF silique wall generated by Cellpose. [file 13007_2022_948_MOESM18_ESM.tif]
