# Supplementary material for: A simple and efficient method to quantify the cell parameters of the seed coat, embryo and silique wall in rapeseed
Source: Plant Methods. 2022 Nov 3;18:117. doi: 10.1186/s13007-022-00948-1 (PMC9632141; doi:10.1186/s13007-022-00948-1)
Supplement: Supplementary file 3 — Additional file 3. ImageJ macros used in this study. [file 13007_2022_948_MOESM3_ESM.docx]

All the macros and algorisms used in this study are listed below.

1. seedsize.ijm

macro "seedsize" {

dir_saving=getDirectory("choose a directory to save"); dir_processing=getDirectory("choose a directory to process");

list=getFileList(dir_processing);

for (i = 0; i < list.length; i++) {

open(list[i]);

title=getTitle();

print(title);

run("8-bit");

setThreshold(0, 100);

run("Analyze Particles...", "size=1-infinity clear include summarize add in_situ");

open(title);

run("From ROI Manager");

saveAs("tiff",dir_saving+title);

close("*");

}

Change the red text to your own parameters.

2. TWS_cell_count.ijm

macro "TWS_cell_count" {

dir_origin=getDirectory("Choose the origin Directory");

dir_saving=getDirectory("Choose a Directory to save");

dir_processing=getDirectory("Choose a Directory to process");

list=getFileList(dir_processing);

for (i = 0; i < list.length; i++) {

open(list[i]);

title=getTitle();

print(title);

run("Make Composite", "display=Grayscale");

run("Split Channels");

run("Close");

run("Threshold...");

setOption("BlackBackground", true);

run("Convert to Mask");

run("Fill Holes");

run("Analyze Particles...", "size=40-300 clear include summarize add in_situ");

open(dir_origin+title);

run("From ROI Manager");

saveAs("tiff",dir_saving+title);

close("*");

}

}

Change the red text to your own parameters.

3. getcellmask.py

import time, os, sys

from urllib.parse import urlparse

import matplotlib.pyplot as plt

import matplotlib as mpl

mpl.rcParams['figure.dpi'] = 300

from cellpose import utils, io

import numpy

os.chdir("E:/my samples")

files = os.listdir("E:/my samples")

from cellpose import models, io

model = models.Cellpose(gpu=False, model_type='cyto')

x=[[0,0],[0,0]]

channels = numpy.repeat(x,[1,len(files)],axis=0)

for chan, filename in zip(channels, files):

    img = io.imread(filename)

    masks, flows, styles, diams = model.eval(img, diameter=None, channels=chan)

  io.save_masks(img, masks, flows, filename, png=False, tif=True, channels=[0,0],suffix='',save_flows=False, save_outlines=False, save_ncolor=False, dir_above=False, in_folders=False,  savedir="E:/sample masks", save_txt=False)

print(filename)

Change the red text to your own parameters.

If you set diameter=None, cellpose will estimate cell diameter automatically. But sometimes this may need to modify, for example, we set diameter=70 for seed coat cells, and acquire high-level performance.

4. celp_mask_sum.ijm

macro " celp_mask_sum" {

dir_origin=getDirectory("Choose the origin Directory");

dir_saving=getDirectory("Choose a Directory to save");

dir_processing=getDirectory("Choose a Directory to process");

list=getFileList(dir_processing);

for (i = 0; i < list.length; i++) {

open(list[i]);

title=getTitle();

pretitle=substring(title, 0, lengthOf(title)-13);

print(pretitle);

run("Set Scale...", "distance=453 known=100 unit=um");

run("8-bit");

run("Find Edges");

setAutoThreshold("Default dark");

setThreshold(0, 0);

run("Analyze Particles...", "size=70-650 circularity=0.40-1.00 exclude clear include summarize add in_situ");

open(dir_origin+pretitle+".tif");

run("From ROI Manager");

saveAs("tiff",dir_saving+pretitle+".tif");

close("*");

}

}

Change the red text to your own parameters.

5. celp_mask_LabelsToROIs.ijm

macro "celp_mask_LabelsToROIs"

{

dir_saving=getDirectory("Choose a Directory to save");

dir_processing=getDirectory("Choose the processing Directory");

list=getFileList(dir_processing);

for (i = 0; i < list.length; i++) {

print(list[i]);

open(list[i]);

title=getTitle();

pretitle=substring(title, 0,lengthOf(title)-13);

print(pretitle);

run("Connected Components Labeling", "connectivity=4 type=[16 bits]");

run("Remove Border Labels", "left right top bottom");

//run("Fill Holes (Binary/Gray)");

run("Label Size Filtering", "operation=Greater_Than size=100");

run("Set Label Map", "colormap=[RGB 3-3-2] background=White shuffle");

saveAs("tiff",dir_saving+pretitle+"_label.tif");

close("*");

}

}

Note: “Fill Holes” will fill the small gap between cells, users should think carefully whether to use it or not.

6. csv_sum.R

#This calculate means of cell parameters in each .csv file in current directory

# This will generate a file SummaryAll.csv in current directory

df1 <- data.frame("sample","CellCount","TotalArea","Area","Perimeter","Feret")

wd <- getwd()

file_name <- list.files(wd)

dir <- paste("./",file_name,sep = "")

n <- length(file_name)

for (i in 1:n) {

m <- substr(file_name[i],nchar(file_name[i])-2,nchar(file_name[i]))

if (m == "csv") {

print(file_name[i])

data <- read.table(dir[i],header = TRUE, sep = ",")

smp <- data$File[1]

count <- data$X[length(data$X)]

total <- sum(data$Area)

area <- mean(data$Area)

perim <- mean(data$Perim.)

feret <- mean(data$Feret)

newline <- c(smp, count, total, area, perim, feret)

df1 <- rbind(df1, newline)

}

}

write.table(df1,"SummaryAll.csv",sep = ",",row.names = F)
