# Supplementary material for: A simple and efficient method to quantify the cell parameters of the seed coat, embryo and silique wall in rapeseed
Source: Plant Methods. 2022 Nov 3;18:117. doi: 10.1186/s13007-022-00948-1 (PMC9632141; doi:10.1186/s13007-022-00948-1)
Supplement: Supplementary file 5 — Additional file 5. Figure S1. Detection of seed coat cells by the TF method. [file 13007_2022_948_MOESM5_ESM.docx]

Additional file 5: Figure S1


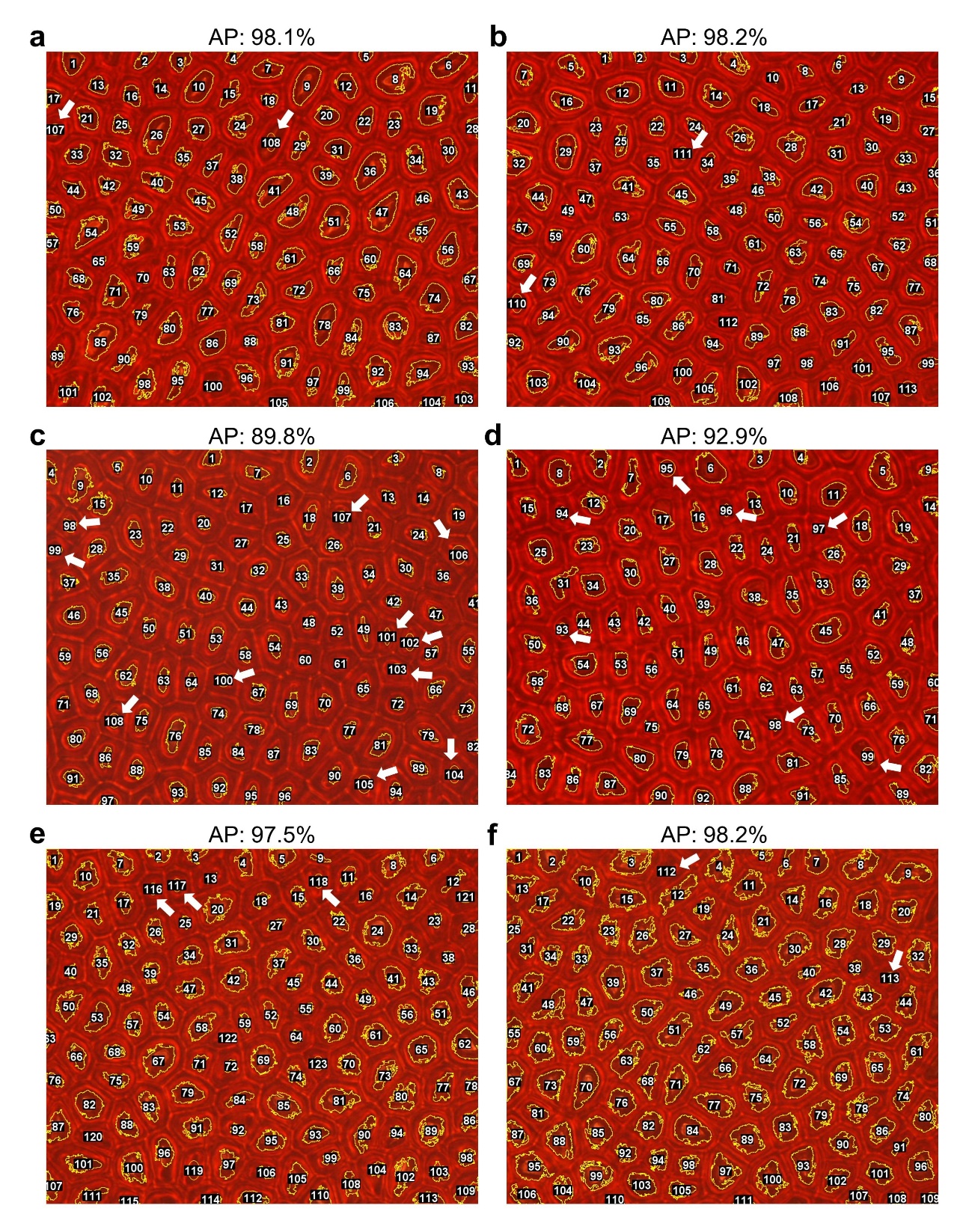


**Additional file 5: Figure S1** Detection of seed coat cells by the TF method.

Most of cells were successfully detected by the TF method in six images. The correctly predicted cells / total cells were 106/108 (**a**), 109/111 (**b**), 97/108 (**c**), 92/99 (**d**) 115/118 (**e**) and 111/113 (**f**), respectively. AP, average precision; The arrows indicate missing cells labeled manually.
