# Supplementary material for: A simple and efficient method to quantify the cell parameters of the seed coat, embryo and silique wall in rapeseed
Source: Plant Methods. 2022 Nov 3;18:117. doi: 10.1186/s13007-022-00948-1 (PMC9632141; doi:10.1186/s13007-022-00948-1)
Supplement: Supplementary file 7 — Additional file 7. Figure S2. Cell quantification of seed coat by the CF method. [file 13007_2022_948_MOESM7_ESM.docx]

Additional file 7: Figure S2


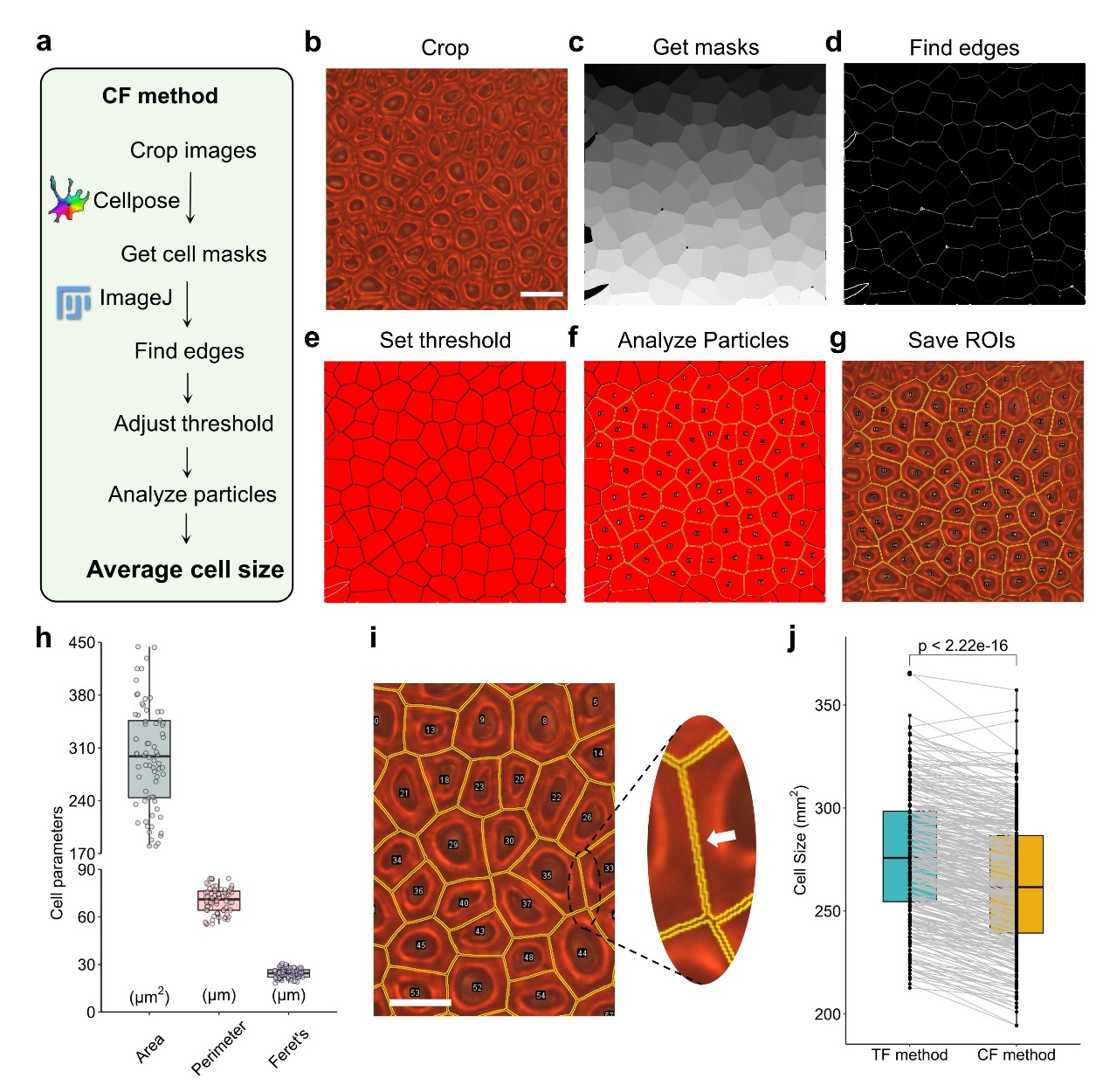


**Additional file 7: Figure S2** Cell quantification of seed coat by the CF method.

**a** Flowchart of the procedure to perform the CF method. **b** A raw cell image was cropped. Bar = 30 μm. **c** The cell masks were acquired using Cellpose. **d** The cell edges were found. **e** A threshold was set. **f** The cells were counted and measured by analyzing particles. **g** The ROIs were added to the original image. **h** The cell parameters were acquired from image (**a**) (n = 70). **i** Enlarged image of segmented cells. The arrow indicates the gaps between adjacent cells. Bar = 20 μm. **j** The average cell size calculated by the TF and CF method, showing a reduction of the CF method cause by systematic error (n=344). The boxplots represent the median and the 25th and 75th quartiles; the whiskers represent the minimum and maximum.
